# Improving bench-to-bedside translation for acute graft-versus-host disease models

**DOI:** 10.1242/dmm.052084

**Published:** 2025-02-28

**Authors:** Brianyell McDaniel Mims, Kathryn L. Furr, Josue Enriquez, Matthew B. Grisham

**Affiliations:** ^1^ Department of Oral Health Sciences, Medical University of South Carolina, Charleston, SC 29425, USA; ^2^ Department of Immunology and Molecular Microbiology, Texas Tech University Health Sciences Center, Lubbock, TX 79423, USA; ^3^ Department of Microbiology and Immunology, University of Gothenburg, Gothenburg 405 30, Sweden

**Keywords:** Anemia, Spleen hypoplasia, Housing temperature, Reduced intensity conditioning, Outbred mice, Microbiota, Conditioning

## Abstract

The transplantation of allogeneic hematopoietic stem cells is a potentially curative treatment for hematological malignancies, inherited blood disorders and immune deficiencies. Unfortunately, 30-50% of patients receiving allogeneic hematopoietic stem cells will develop a potentially life-threatening inflammatory disease called acute graft-versus-host disease (aGVHD). In patients with aGVHD, graft-associated T cells, which typically target the skin, intestinal tract and liver, can also damage the lungs and lymphoid tissue. Damage to lymphoid tissue creates prolonged immunodeficiency that markedly increases the risk of infections and bleeding, resulting in considerable morbidity and mortality. Although mouse models of aGVHD have been instrumental to our understanding of this condition's pathogenesis, translation of preclinical data into new and more effective treatments for human disease has been limited for reasons that remain to be fully understood. However, evidence suggests that factors associated with mouse models of aGVHD likely contribute to these unsatisfactory results. In this Review, we identify and discuss the specific factors inherent to mouse models of aGVHD that may limit the translation of preclinical data to patient treatment, and suggest how to improve the translatability of these models.

## Teaser

**Summary:** In this Review, we discuss specific factors inherent in mouse models of acute graft-versus-host disease that may limit translation of preclinical data into more effective patient treatment, as well as provide suggestions to improve the translatability of these models.

## Introduction

**Fig. 1.**
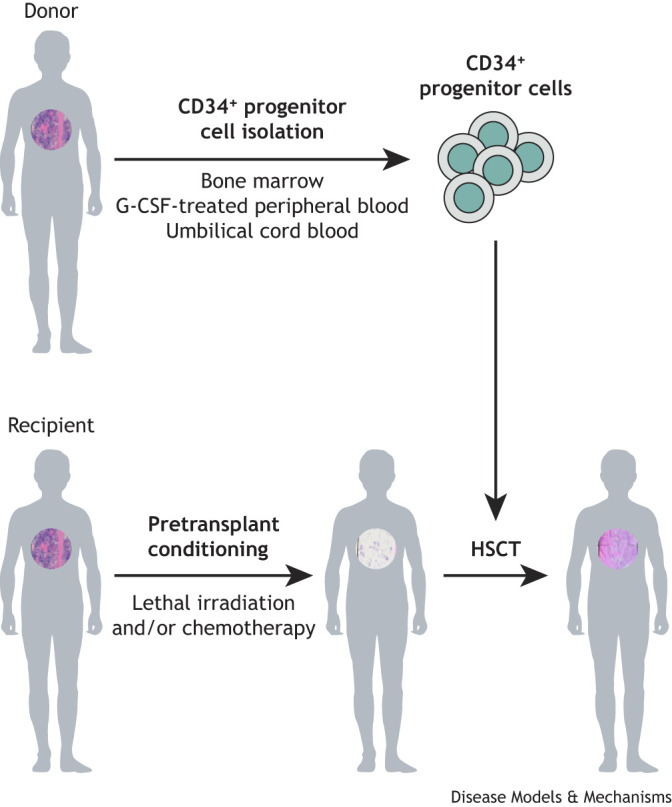
**Hematopoietic stem cell transplantation (HSCT).** CD34^+^ progenitor cells are isolated from donor bone marrow, granulocyte-colony stimulating factor (G-CSF)-treated peripheral blood or umbilical cord blood prior to pretransplant conditioning of the recipient. Recipients then receive myeloablative, pretransplant conditioning to suppress graft rejection and provide space in the bone marrow for donor progenitor cells to engraft and proliferate. If the patient suffers from a hematologic malignancy, pretransplant conditioning will eradicate residual tumor cells as well. Donor CD34^+^ progenitor cells are then infused into the recipient. Circular images within the individuals are representations of bone marrow cellularity in donors and recipients at different times during HSCT. Note the loss of bone marrow cellularity in the recipient following myeloablative, pretransplant conditioning. Engraftment of healthy donor progenitor cells within the lymphopenic recipient results in the differentiation and expansion of all the cellular elements of the blood.

Hematopoietic stem cell transplantation (HSCT) is a potentially life-saving treatment for refractory/relapsing hematological malignancies (see Glossary, Box 1), severe blood diseases and primary immunodeficiency diseases (Blazar et al., 2020; Shimoni et al., 2016; Zeiser and Blazar, 2017a; Hall and de Zoeten, 2024; Rao et al., 2007; Nguyen et al., 2015). This procedure involves the transplantation of CD34^+^ progenitor cells (Box 1) derived from three main sources: healthy donor bone marrow, peripheral blood from granulocyte colony-stimulating factor (G-CSF; also known as CSF3)-treated donors or umbilical cord blood (Fig. 1). Before the infusion of these progenitor cells, recipients undergo pretransplant conditioning using whole-body radiation and/or myeloablative chemotherapy (Box 1), which destroys the bone marrow (BM), thereby creating immunological space for engraftment of donor cells (Fig. 1), as well as eliminates cancer cells present within recipients. Patients are then infused with the donor progenitor cells, which engraft, differentiate and proliferate into all of the cellular elements of blood. A recent update from the Center for International Bone and Marrow Transplant Research reported that a total of 23,535 individuals in the USA received HSCT (Phelan et al., 2022). Of these, 14,236 patients received their own (i.e. autologous) progenitor cells, while the other 9299 individuals received allogeneic progenitor cells from related or unrelated donors. For many indications, the preferred hematopoietic stem cell (HSC) donor is a human leukocyte antigen (HLA)-matched sibling (Box 1). However, the chance of finding such a donor varies widely, ranging from 13% to 51% depending on ethnic background and age (Spellman, 2022). The next best option is an HLA-matched unrelated donor, the availability of which in the USA ranges from 29% for African American patients to 78% for white non-Hispanic patients (Spellman, 2022). In the absence of these HLA-matched donors, patients may receive HSCs from either a related or unrelated haploidentical donor who shares ∼50% of the HLA alleles with the recipient. Stem cells from these allogeneic donors can then be used for >95% of all patients in need of HSCT (Baumeister et al., 2020; Spellman, 2022).

**Box 1. Glossary**

**Adenocarcinoma:** a malignant (cancerous) tumor that arises from glandular cells in various organs such as the colon, lungs, prostate and pancreas.

**Adenoma:** a type of benign (non-cancerous) tumor that originates in the glandular tissue of organs.

**Anti-CTLA-4:** this monoclonal antibody is an immune checkpoint inhibitor that targets and blocks cytotoxic T-lymphocyte antigen-4 (CTLA-4), a receptor found on T cells. CTLA-4 plays a crucial role in downregulating immune responses, helping to prevent autoimmune diseases by limiting T-cell activation.

**Bacteremia:** refers to the presence of bacteria in the bloodstream. Because blood is normally sterile, the presence of bacteria in this environment can cause serious infections and systemic complications.

**BALB/c (H-2^d^) mice:** these mice are a specific substrain of BALB/c mice that are characterized by their genetic background and the H-2^d^ haplotype, which is part of the major histocompatibility complex (MHC) in mice. The H-2 region is crucial for immune system function**,** particularly in the context of antigen presentation and immune responses. They are a widely used strain of inbred laboratory mice that have become one of the most popular models for immunological and cancer research.

**C57BL/6 (H-2^b^) mice:** C57BL/6 mice that are one of the most widely used inbred strains of laboratory mice, known for their genetic uniformity and robust immune response, making them a standard model in many fields of biomedical research. Their H-2^b^ haplotype is part of their MHC.

**CByJ.SJL(B6)-*Ptprc^a^*/J CD45.1 (H-2^d^) mice:** these mice are CD45.1 congenic mice that are derived from the C57BL/6 (B6-H-2^d^) background. They possess a specific genetic modification in the *Ptprc* gene, which encodes the CD45 protein. The CD45.1 allele allows for the distinction of donor and recipient immune cells, making these mice an invaluable tool for studying immune reconstitution, graft-versus-host disease (GVHD) models and stem cell biology.

**CD34^+^ progenitor cells:** these cells are a type of stem cell found in the bone marrow and in peripheral blood**.** Surface expression of CD34 is used to identify (and isolate) hematopoietic stem cells**.**

**CD4^+^ and CD8^+^ T cells:** these are two subsets of T lymphocytes that play crucial roles in adaptive immunity. Interaction of CD4^+^ T cells with antigen-presenting cells (e.g. dendritic cells, macrophages and B cells) enhances their ability to kill pathogenic microorganisms, whereas CD8^+^ cytotoxic T cells interact with and kill cancer cells or viral-infected cells.

**CD4^+^CD25^−^ conventional T cells:** a subset of CD4^+^ T cells that are involved in the activation and regulation of immune responses. CD4^+^CD25^−^ conventional T cells play helper and effector roles, promoting inflammation, immune activation and pathogen clearance.

**CD4^+^CD25^+^Foxp3^+^ regulatory T cells:** these cells are involved in immune tolerance and help to prevent autoimmune diseases by suppressing the activity of other immune cells that could attack the body's own tissues. They play an important role in maintaining immune homeostasis and preventing excessive inflammation.

**CD45-ADC-conditioned mice:** mice that have been treated with an antibody-drug conjugate (ADC) targeting the CD45 antigen to deplete immune cells that express CD45 residing in the bone marrow and other lymphoid tissue. In the future, this type of conditioning protocol could be used in allogeneic bone marrow transplantation.

**Commensal microbiota:** the term ‘commensal’ comes from the Latin word ‘commensalis’, meaning ‘sharing a table’, reflecting the idea that these microorganisms thrive within the host's environment without directly benefiting or harming it. Commensal microbiota include bacteria, fungi, viruses and other microbes, which inhabit the gut, skin, mouth, respiratory and urogenital tracts.

**Conventional housing conditions:** conditions in which mice are housed in cages with wire mesh tops, thereby exposing them to ambient room air. Although care is taken to avoid the introduction of infectious agents, conventional housing does not necessarily require the strict protective measures that are used for specific pathogen-free (SPF) housing. Thus, the microbiome of conventionally housed mice may be quite different from that of SPF animals, leading to greater variability in experimental outcomes.

**Cyclosporine A:** a powerful immunosuppressive drug that plays a crucial role in preventing GVHD, organ rejection in transplant recipients and managing autoimmune diseases. Its ability to block T-cell activation makes it an essential therapeutic agent.

**Cytopenia:** a reduction in the numbers of blood cells, which can be caused by a variety of conditions, including bone marrow disorders, infections, toxins and certain medications.

**Donor chimerism:** a condition in which a recipient's body contains a mixture of both host (recipient-derived) and donor-derived cells. This condition is observed following hematopoietic stem cell transplant, bone marrow transplant or organ transplant, where the transplanted stem cells or tissue from the donor start to engraft and contribute to the recipient's immune system and tissues.

**Dysbiosis:** an imbalance or disruption in the normal microbial community of the gut. Under healthy conditions, these microbes play a crucial role in digestion, immunity and maintaining the integrity of the intestinal lining. Disruption of the gut microbiota may lead to health problems.

**Fanconi anemia:** a complex, genetic disorder characterized by bone marrow failure, congenital abnormalities and an increased risk of cancers, particularly leukemia. Currently, there is no cure for Fanconi anemia; however, bone marrow transplantation offers the potential for long-term survival.

**Feral mice:** wild, non-domesticated rodents that live in natural or human-modified environments. They differ from laboratory mice in terms of behavior, health, lifespan and reproductive habits.

**Granzymes:** a family of serine proteases, primarily produced by cytotoxic immune cells such as natural killer (NK) cells and CD8^+^ cytotoxic T lymphocytes**.** They play a crucial role in the immune system's ability to eliminate infected, cancerous or otherwise abnormal cells. Granzymes are stored in cytoplasmic granules of these immune cells and are released upon T-cell activation.

**Human leukocyte antigen (HLA)-matched sibling:** a brother or sister who shares the same set of HLA molecules, which are key components of the immune system, particularly involved in immune response regulation. These molecules are encoded by a group of genes located on chromosome 6, and they play a central role in the body's ability to distinguish between self and non-self cells.

**Interleukin (IL)-2 receptor common γ chain:** a vital subunit shared by several important cytokine receptors that plays a central role in immune cell development, activation and function.

***Listeria monocytogenes*:** a pathogenic bacterium that is primarily transmitted through contaminated food and capable of causing the infection listeriosis. The infection can be serious, especially for pregnant women, newborns, older-age and immunocompromised individuals.

**Lymphopenia:** a condition marked by low levels of lymphocytes in the blood that can result from a variety of causes, including infections, autoimmune diseases, GVHD, medications or bone marrow disorders. It can lead to increased susceptibility to infections and other immune system-related complications.

**Lymphotoxin-α:** a critical cytokine in the immune system, involved in the development of lymphoid organs, regulation of immune responses and inflammation. It interacts with the lymphotoxin-β receptor to promote immune cell activation, tissue remodeling and inflammation.

**Major histocompatibility complex (MHC):** a set of proteins that play a crucial role in the immune system by presenting antigens to T cells, which are necessary for the initiation of adaptive immune responses. MHC Class I molecules present intracellular antigens to CD8^+^ cytotoxic T cells**,** whereas MHC Class II molecules present extracellular antigens to CD4^+^ T-helper cells. Although MHC diversity is critical for pathogen recognition, mismatched MHC molecules can lead to transplant rejection or GVHD.

**Methotrexate:** a chemotherapeutic agent and an immunosuppressive drug used in the treatment of certain types of cancer, as well as alloimmune and autoimmune diseases. It belongs to the class of drugs called antimetabolites and works by interfering with the rapidly dividing cells.

**MHC mismatch:** involves a significant difference between the donor and recipient's MHC molecules, particularly in the Class I and Class II regions. This mismatch can trigger strong immune responses because the immune system will recognize the donor tissue as ‘foreign’. Infusion of MHC-mismatched hematopoietic stem cells into a recipient will induce GVHD.

**Minor histocompatibility antigen (miHA):** a type of peptide produced from proteins encoded by polymorphic, non-MHC-encoded genes. These peptides are presented by MHC I or MHC II molecules.

**miHA mismatch:** this type of mismatch generally leads to more subtle immune responses. However, these mismatches are still capable of playing a significant role in graft rejection or GVHD.

**Myeloablative chemotherapy:** a high-dose chemotherapy and/or irradiation treatment used to destroy bone marrow to prepare the body for hematopoietic stem cell transplantation**.**

**Myelodysplastic syndrome or myelodysoplasia:** a group of bone marrow disorders characterized by ineffective production of blood cells, leading to anemia, neutropenia and thrombocytopenia. These diseases may be primary (idiopathic) or secondary to previous treatments such as chemotherapy or radiation.

**NSGW41 and NBSGW mice:** NSGW41 (NOD.Cg-*Kit^W-41J^ Prkdc^scid^ Il2rg^tm1Wjl^*/WaskJ) and NBSGW (NOD.Cg-*Kit^W-41J^ Tyr*^+^ *Prkdc^scid^ Il2rg^tm1Wjl^*/ThomJ) mice contain a specific mutation in the *Kit* gene that increases immunological space in the bone marrow, thereby enhancing engraftment of human hematopoietic stem cells without γ-radiation of the mice.

**Pan T cell-depleting antibodies:** these antibodies are powerful immunosuppressive agents that target and deplete T cells by binding to broad T-cell markers such as CD3. They used in clinical settings such as organ transplantation, autoimmune diseases and GVHD to reduce the activity of harmful T cells.

**Perforin:** this protein plays a crucial role in the immune responses to infected cells, tumor cells and cells involved in autoimmune or alloimmune reactions. Perforin is produced by CD8 cytotoxic T cells and NK cells, which eliminate harmful cells. Perforin works by creating pores in the membrane of target cells, allowing other molecules, such as granzymes, to enter and induce cell death.

**Recombination activating gene-2 (*RAG2*):** a vital gene involved in V(D)J recombination, which is the process that generates the diversity of T-cell receptors and antibodies required for immune system function. Deficiencies or mutations in *RAG2* can lead to severe combined immunodeficiency disease.

**Refractory/relapsing hematological malignancies:** these malignancies refer to blood cancers (e.g. leukemia, lymphoma and multiple myeloma) that either do not respond to treatment (refractory) or initially respond to treatment but later return (relapsing). These conditions are particularly challenging because they are resistant to standard therapies, leading to poorer outcomes for patients.

**Rhesus monkeys:** *Macaca mulatta* are a species of monkeys native to India, China and Southeast Asia. They are one of the most well-known and extensively studied primate species that have been widely used in biomedical research owing to their genetic similarity to humans and have contributed to advancements in medicine, including vaccine development and human immunodeficiency virus (HIV) research.

**Tacrolimus:** an immunosuppressive medication primarily used to prevent organ/tissue rejection following transplantation and to treat various autoimmune diseases. It works by inhibiting the activity of the immune system, specifically targeting T-cell activation, to reduce the likelihood of the body rejecting a transplanted tissue.

**T-helper (Th)1 and Th17 cells:** Th1 cells produce interferon-γ (IFNγ) and other cytokines that activate macrophages and enhance the response against intracellular pathogens such as viruses and certain bacteria. Th17 cells produce IL-17, which promotes the recruitment of neutrophils and other inflammatory cells to sites of infection or injury. Th17 cells are important in the defense against fungal infections and some bacterial pathogens. Th17 responses have been implicated in certain autoimmune diseases (e.g. rheumatoid arthritis and multiple sclerosis).

**Thrombocytopenia:** characterized by a low platelet count in the blood. Platelets (thrombocytes) are produced in the bone marrow and play a critical role in blood clotting and wound healing. Low platelet numbers can lead to increased risk of bleeding and bruising.

**Tumor necrosis factor receptor superfamily member 6 (FAS)** this cell surface receptor (also known as CD95) plays an important role in regulating programmed cell death, i.e. apoptosis. Binding of FAS to its ligand FASL triggers series of intracellular reactions, culminating in the death of the target cell.

**Unfractionated splenocytes:** a single-cell mixture of all the immune cells present in the spleen. Unfractionated splenocytes are often used in immunology and transplantation studies to investigate immune responses.

Unfortunately, 30-70% of allogeneic HSCT patients will develop a potentially life-threatening, inflammatory disease called acute graft-versus-host disease (aGVHD) (Zeiser and Blazar, 2017a; Hill et al., 2021). This tissue-destructive inflammatory disease usually occurs within 1-3 months following allogeneic HSCT, although it can occur at later timepoints. That said, important new clinical studies demonstrate that post-transplant administration of high-dose cyclophosphamide allows for the use of HLA-disparate donors in HSCT (Shaffer et al., 2024; Shaw et al., 2021; Bolanos-Meade et al., 2023). These clinical studies were based, in part, on the important preclinical findings that cyclophosphamide administration limits aGVHD in mouse models of allogeneic HSCT (O'Donnell and Jones, 2023). In a 2024 study, Shaffer et al. (2024) reported that the 3-year overall survival and aGVHD-free survival of patients who received HLA-mismatched unrelated donor transplants together with post-transplant cyclophosphamide were essentially the same as those of patients who received HLA-matched unrelated donor transplants and cyclophosphamide treatment. This multi-ethnic study [which included Hispanic and non-Hispanic whites, African Americans, Asians, American Indians, Native Hawaiians and Pacific Islanders diagnosed with different leukemias or myelodysplastic syndrome (Box 1)] demonstrated that administration of post-transplant cyclophosphamide could greatly expand the use of HSCT for patients from most ethnic and racial backgrounds.

Both experimental and clinical data demonstrate that allogeneic T cells are the major immune cell type that initiates aGVHD (Hess et al., 2021; Hill et al., 2021; Zeiser and Blazar, 2017a). Although these lymphocytes typically target the skin, gut and liver, they can also damage the lungs and lymphoid tissue (Malard et al., 2023; Zeiser and Blazar, 2017a; Zeiser and Teshima, 2021; Teshima and Hill, 2021). Importantly, allogenic T cell-mediated damage to primary lymphoid tissue (BM, thymus) and secondary lymphoid tissue (lymph nodes, spleen) can result in severe lymphopenia (Box 1), thrombocytopenia (Box 1) and anemia (Muskens et al., 2021; Shono et al., 2014, 2010; Chewning et al., 2013; McDaniel Mims et al., 2019; Cuthbert et al., 1995; Shahin et al., 2017; Zeiser and Teshima, 2021; Enriquez et al., 2023). The immunodeficiency caused by aGVHD markedly increases the risk of infections and bleeding, and of morbidity and mortality, accounting for ∼30% of patient deaths (Muskens et al., 2021; Shono et al., 2014, 2010). Based upon early preclinical studies that demonstrated the ability of corticosteroids to reduce aGVHD in mouse models (You-Ten et al., 1995), high doses of corticosteroids are now used as the first line of defense for patients undergoing HSCT. Nevertheless, 40% of patients with aGVHD do not respond to this therapy, with few second-line therapies available (Malard et al., 2023). Six-month survival for steroid-resistant patients is ∼50%, with survival reduced to 30% in patients surviving >2 years (Zeiser et al., 2023). Although out of the scope of this Review, it is worth mentioning that allogeneic HSCT can also induce a chronic form of the disease called chronic graft-versus-host disease (cGVHD), which affects 30-70% of patients who survive for 5-6 months following this type of transplant (Hill et al., 2021; Zeiser and Blazar, 2017b). cGVHD is a heterogeneous disease that is immunologically different from aGVHD as it can affect any tissue, resulting in fibrotic disease of the face, eyes, musculoskeletal system, genitals and/or joints (Hill et al., 2021; Zeiser and Blazar, 2017b).

## Animal models of aGVHD

Much of our early understanding of aGVHD came from preclinical studies using canine models of allogenic HSCT. Data generated from these large-animal models more than 50 years ago established some of the first HSCT protocols in patients (Teshima and Hill, 2021; Thomas et al., 1975a,b). For example, the prophylactic administration of both cyclosporine A (Box 1) [or tacrolimus (Box 1)] and methotrexate (Box 1) together with pan T cell-depleting antibodies (Box 1) was first proven to be effective in attenuating aGVHD in canine models of HSCT (Deeg et al., 1982; Kolb et al., 1979). These groundbreaking studies culminated in Dr E. Donnall Thomas receiving the Nobel Prize in Physiology or Medicine in 1990. Although canine models of disease continue to be used, the costs of inbred dogs, together with the costs for housing/care in an animal care facility, are far greater than those of rodents. Because the vast majority of all preclinical studies investigating the immunopathogenesis of aGVHD use mouse models, this Review will focus on the different mouse models of disease.

## Allogeneic mouse models

The use of inbred allogeneic HSCT in mice has been instrumental in delineating several of the key aspects of aGVHD pathogenesis as well as providing investigators with potential targets for drug discovery. The major advantages of using these inbred mouse models are their relatively low cost and reproducibility, which have allowed investigators to examine specific pathogenetic mechanisms via the use of a wide variety of readily available pharmacological and immunological reagents (Table 1) (Schroeder and DiPersio, 2011; Stolfi et al., 2016; Zeiser and Blazar, 2016, 2017a; Hess et al., 2021; Markey et al., 2014; Patel et al., 2022; Reddy et al., 2008). Data generated from numerous aGVHD mouse studies have identified and characterized the sequences of events that lead to the development of aGVHD.

**Table 1.** Representative mouse models of acute graft-versus-host disease

Model	Donor strain	Recipient strain	Donor cells	Outcomes	References
Conditioning: lethal irradiation					
MHC mismatched	C57BL/6 (H-2^b^)	BALB/c (H-2^d^)	BM+splenocytes	Lethal disease with skin involved	van Leeuwen et al., 2002
	C57BL/6 (H-2^b^)	B6.C-H-2^bm12^	BM+CD4^+^ T cells	Lethal disease	Sprent et al., 1986
	C57BL/6 (H-2^b^)	B6.C-H-2^bm1^	BM+CD8^+^ T cells	Lethal disease	Sprent et al., 1986
	C57BL/6 (H-2^b^)	B6D2/F1 (H-2^b/d^)	BM+T cells	Lethal disease involving liver and intestine	Blaser et al., 2005
	C3H/Hej (H-2^k^)	C57BL/6 (H-2^b^)	BM+splenocytes	Lethal disease	Blazar et al., 1991
					
miHA mismatched	B10.BR (H-2^k^)	BALB/k (H-2^k^)	BM+splenocytes	Lethal disease involving skin, lung and/or liver	Fanning et al., 2009
	B10.D2 (H-2^d^)	BALB/c (H-2^d^)	BM+splenocytes	Lethal disease involving skin, lung and/or liver	Eyrich et al., 2005
	B10 (H-2^b^)	BALB/b (H-2^b^)	BM+splenocytes	Lethal disease involving skin, lung and/or liver	Kaplan et al., 2004
	C57BL/6 (H-2^b^)	BALB/b (H-2^b^)	BM+T cells	Lethal disease involving skin, lung and/or liver	Zhang et al., 2005
Conditioning: RIC					
MHC mismatched	B6.C-H-2^bm12^	C57BL/6 (H-2^b^)	CD4^+^CD25^−^ T cells	Lethal disease involving BM failure and spleen aplasia	Enriquez et al., 2023
	C57BL/6 (H-2^b^)	BcB6F (H-2^b/d^)	Splenocytes	Lethal disease involving BM failure	Arieta Kuksin et al., 2015
					
miHA mismatched	C57BL/6 (H-2^b^)	BALB/b (H-2^b^)	BM+splenocytes	Lethal disease involving BM damage	Shahin et al., 2017
	C57BL/6 (H-2^b^)	B6D2/F1 (H-2^b/d^)	Lymph node cells	Lethal disease involving BM failure	Chen et al., 2004
Conditioning: NK cell depletion					
MHC mismatched	BALB/c (H-2^d^)	C57BL/6 NK cell-depleted *Rag1*^−/−^ (H-2^b^)	BM+splenocytes	Lethal disease involving liver, skin and intestine	Nalle et al., 2014
	BALB/c (H-2^d^)	C57BL/6 NK cell-depleted *Rag1*^−/−^ (H-2^b^)	CD4^+^CD25^−^ T cells	Lethal disease involving liver, skin and BM	McDaniel Mims et al., 2019
	C57BL/6 (H-2^b^)	BALB/c *Rag2*^−/−^*Il2rg*^−/−^ (H-2^d^)	CD4^+^CD25^−^ T cells	Lethal disease involving skin and BM	McDaniel Mims et al., 2019

BM, bone marrow; MHC, major histocompatibility complex; miHA, minor histocompatibility antigen; NK, natural killer; *Rag1*^−/−^, recombination activating gene-1 deficient; *Rag2*^−/−^*Il2rg*^−/−^, recombination activating gene-2 and interleukin-2 receptor common γ gene double-deficient; RIC, reduced intensity conditioning. ‘H-2’ with superscripted characters indicates the MHC H-2 haplotype.

## Phases of disease development

### Initiation phase

**Fig. 2.**
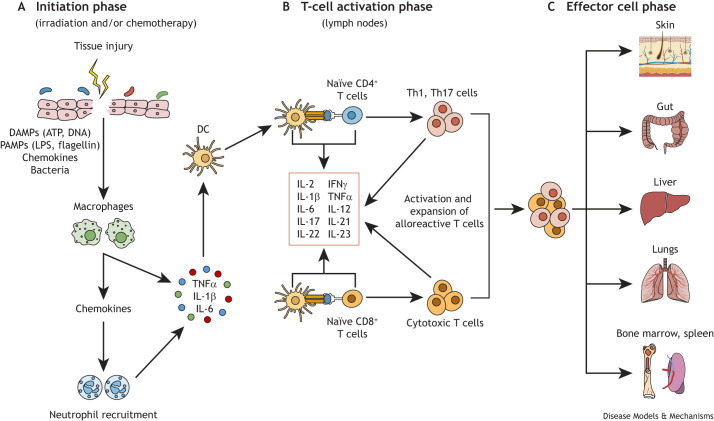
**Proposed immunopathogenesis of acute graft-versus-host disease (aGVHD).** (A) Initiation phase: pretransplant conditioning protocols damage the epithelia of tissues that are in contact with commensal microbiota (e.g. skin and gut), resulting in the translocation of bacteria and their products into the tissue and upregulation of multiple pro-inflammatory mediators, such as damage-associated molecular patterns (DAMPs), pathogen-associated molecular patterns (PAMPs), chemokines and chemokine receptors. Inflammatory mediators activate tissue macrophages to produce additional inflammatory cytokines and chemokines that promote infiltration and activation of innate immune cells (e.g. neutrophils, monocytes). Activation of these myeloid cells increases further the ‘cytokine storm’ that activates tissue-residing dendritic cells (DCs). Once activated, DCs migrate from the tissue into the lymphatic vessels, where they are transported to the draining lymph nodes. (B) T-cell activation phase: interaction of recipient DCs with donor-derived alloreactive CD4^+^ and CD8^+^ T cells drives the activation, differentiation and expansion of these pathogenic effector cells [e.g. CD4^+^ T-helper (Th)1 and Th17 cells and CD8^+^ cytotoxic effector cells]. All three groups of alloreactive T cells exit the lymph nodes via the efferent lymphatics and enter the systemic circulation, in which they traffic to the different target tissues. (C) Effector cell phase: CD8^+^ cytotoxic T cells damage host cells via the release of tumor necrosis factor-α (TNFα), interferon-γ (IFNγ), perforin, granzymes and interaction of FAS with FAS ligand, whereas alloreactive Th1 effector cells induce cell and tissue injury directly via their production of IFNγ, TNFα, interleukin (IL)-2 and lymphotoxin-α, or indirectly via recruitment and/or activation of innate immune cells. Although Th1 responses are considered the primary driver of aGVHD, Th17 cells also play a role in inflammatory tissue damage by promoting the recruitment and activation of additional inflammatory cells. ATP, adenosine triphosphate; DNA, deoxyribonucleic acid; LPS, lipopolysaccharide.

Intravenous administration of major histocompatibility complex (MHC; Box 1)-mismatched (Box 1) HSCs induces aGVHD that occur in three major phases: the initiation phase, the activation phase and the effector cell phase (Fig. 2) (Malard et al., 2023; Schroeder and DiPersio, 2011; Blazar et al., 2012). The initiation phase (Fig. 2A) begins following myeloablative pretransplant conditioning of the recipient (Fig. 1). The large majority of mouse models of aGVHD use lethal total-body irradiation (TBI) to ablate the recipient's BM prior to engraftment with allogeneic BM supplemented with a source of allogeneic T cells, such as unfractionated splenocytes (Box 1) or isolated CD4^+^ and/or CD8^+^ T cells (Box 1) (Schroeder and DiPersio, 2011). TBI is a toxic conditioning protocol that damages multiple tissues, which respond with the production of numerous inflammatory mediators, including damage-associated molecular patterns, cytokines, chemokines and chemokine receptors (Khuat et al., 2021; Hill and Koyama, 2020; Blazar et al., 2020; Zeiser et al., 2016; Castor et al., 2012; Mapara et al., 2006; Chewning et al., 2013; Dudakov et al., 2017; Schroeder and DiPersio, 2011; Shono et al., 2014, 2010; Stolfi et al., 2016; Zeng et al., 2017). In addition, TBI injures epithelia of tissues in direct contact with commensal microbiota (Box 1) (e.g. skin and gut), resulting in the translocation of bacteria and their products, which induces microbe-associated molecular patterns and multiple pro-inflammatory mediators, as described above (Blazar et al., 2012; Schwab et al., 2014; Staffas et al., 2017; Zeiser and Blazar, 2017a; Malard et al., 2023). Although the number of commensal bacteria within the healthy liver is relatively low compared to the numbers in the skin and gut, bacterial counts can increase dramatically following TBI owing to the transportation of whole bacteria (and their products) from the damaged gut to the liver via the portal vein (Malard et al., 2023). The resulting inflammatory environment within the different tissues leads to the infiltration and activation of innate immune cells, such as polymorphonuclear leukocytes, monocytes and tissue-residing dendritic cells (DCs). Once activated, DCs migrate from the tissue into the lymphatic vessels, where they are transported into tissue-draining lymph nodes, resulting in initiation of the activation phase (Malard et al., 2023; Schroeder and DiPersio, 2011; Blazar et al., 2012).

### Activation phase

This phase is characterized by the upregulation of MHC I and MHC II on activated DCs, which facilitates their binding to, and activation of, donor-derived, alloreactive CD4^+^ and/or CD8^+^ T cells (Fig. 2B). Activation of T cells drives the differentiation and expansion of disease-producing effectors cells that include T helper (Th)1 and Th17 cells (Box 1), as well as cytotoxic CD8^+^ T cells. All three groups of alloreactive T cells then exit the lymph nodes via the efferent lymphatics and enter the systemic circulation, in which they traffic to different target tissues to initiate the effector cell phase (Malard et al., 2023; Schroeder and DiPersio, 2011; Blazar et al., 2012).

### Effector cell phase

During this phase, cytotoxic CD8^+^ T cells damage host cells by releasing tumor necrosis factor-α (TNFα; also known as TNF), interferon-γ (IFNγ), perforin (Box 1) and granzymes (Box 1), and via the interaction of FAS (Box 1) with FAS ligand (Fig. 2C). Alloreactive Th1 effector cells can induce cell and tissue injury by producing IFNγ, TNFα, interleukin (IL)-2 and lymphotoxin-α (Box 1). These inflammatory cytokines can also promote inflammatory tissue damage indirectly via their ability to recruit and/or activate innate immune cells (e.g. macrophages, neutrophils, DCs), as well as activate additional cytotoxic CD8^+^ T cells (Blazar et al., 2012; Hill and Koyama, 2020; Malard et al., 2023; Zeiser and Blazar, 2016, 2017a; Zeiser et al., 2016). Although Th1 responses are considered to be the primary driver of aGVHD, IL-17 (also known as IL17A)-producing T cells also play a significant role by promoting the recruitment of neutrophils and amplifying the inflammatory response (Carlson et al., 2009; Kappel et al., 2009) (Fig. 2C).

## Xenogeneic mouse models

There is no question that mouse models of aGVHD have greatly advanced our understanding of disease pathogenesis. However, it has become increasingly apparent that alloimmune responses in mice may differ substantially from those in humans. Indeed, the translation of promising therapeutics that were found to be effective in conventional mouse models of aGVHD has been disappointing in clinical studies (see ‘Therapeutic intervention in mouse models of aGVHD’ section) (Stolfi et al., 2016; Zeiser and Blazar, 2016; Rayasam and Drobyski, 2021; Enriquez et al., 2020; Blazar et al., 2020; Hess et al., 2021; Kennedy et al., 2021). In an attempt to enhance translation of preclinical data to patient treatment, investigators have developed a variety of humanized mouse models of xenogenic graft-versus-host disease (xGVHD) (Table 2) (Chupp et al., 2024; Elhage et al., 2022; Hess et al., 2021; Hess et al., 2020; Ye and Chen, 2022). One of the best-characterized mouse models of xGVHD demonstrates that intravenous administration of human peripheral blood mononuclear cells (PBMCs) into mildly irradiated, nonobese diabetic-severe combined immunodeficient mice lacking the IL-2 receptor common γ chain (Box 1) (NSG mice) induces lethal xGVHD involving the liver, lung and skin (Brehm et al., 2019; King et al., 2009) (Table 2). It should be noted that PBMCs contain T cells, B cells and CD34^+^ progenitor cells. Additional studies by Brehm et al. (2019) using this model reported that when NSG mice were rendered devoid of MHC I and MHC II, engraftment of human T cells failed to induce aGVHD. Furthermore, onset of disease in xGVHD models is associated with enhanced production of several human T cell-derived cytokines and growth factors (Kawasaki et al., 2018). Taken together, these studies demonstrated that human T-cell receptors are capable of recognizing murine MHC I and MHC II, resulting in the activation, polarization and expansion of pathogenic T cells (Brehm et al., 2019). Current evidence shows that the large majority of human T cells derived from xenogeneic mice are Th1 effector cells (Hess et al., 2021, 2020). This is not surprising given the fact that circulating levels of human IL-12 are increased in xGVHD mice (Kawasaki et al., 2018). Another important observation made by investigators using xGVHD models is that tissue inflammation may occur in the absence of pretransplant conditioning, suggesting that nonspecific tissue damage and the consequent cytokine storm are not required for induction of disease (Table 2) (Hess et al., 2021). However, it is highly likely that inflammatory mediators produced during xenogeneic T-cell activation modulate the incidence, progression and severity of disease (Hess et al., 2021).

**Table 2.** Representative xenogeneic mouse models of acute graft-versus-host disease

Donor cells	Recipient	Conditioning	Outcomes	References
Human PBMCs	NSG	Sublethal irradiation (2 Gy)	Lethal disease involving spleen, liver, lung, intestine and bone marrow	King et al., 2009
Human PBMCs	NSG	Sublethal irradiation (2 Gy)	Lethal disease involving liver, lung, skin and kidneys	Chun et al., 2020
Human PBMCs	NSG	Sublethal irradiation (2.5 Gy)	Lethal disease involving lung	Bruck et al., 2013
Human PBMCs	NSG	No conditioning	Lethal disease involving liver, lung and skin	Cuthbertson et al., 2020; Geraghty et al., 2017
Human PBMCs	NOG	Sublethal irradiation (2.5 Gy)	Lethal disease involving liver, lung and kidneys	Ito et al., 2009
Human PBMCs	NSG	No conditioning	Lethal disease involving liver, lung and skin	Brehm et al., 2019
Human PBMCs	NPG	Sublethal irradiation (2.5 Gy)	Lethal disease involving spleen, liver, lung and intestine	Gao et al., 2021
Human BM-MNCs	NBSGW	No conditioning	Lethal disease involving liver, kidney and salivary glands	Hess et al., 2020

BM-MNCs, bone marrow mononuclear cells; NBSGW, NOD.Cg-*Kit^W-41J^ Tyr*^+^ *Prkdc^scid^ Il2rg^tm1Wjl^*/ThomJ mice; NOG, NOD.Cg-*Prkdc^scid^ Il2rg^tm1Sug^* mice; NPG, NOD-*Prkdc^scid^ Il2rg*^null^ mice; NSG, NOD.Cg-*Prkdc^scid^ Il2rg^tm1Wjl^*/SzJ mice; PBMCs, peripheral blood mononuclear cells.

As with any animal model of human disease, xGVHD has a few noteworthy limitations. For example, although the PBMCs→NSG model is characterized by expansion of xenoreactive human T cells, development and/or function of human B cells, monocytes, macrophages and natural killer (NK) cells is poor (Rongvaux et al., 2013). In addition, these xenogeneic mice maintain their murine innate immune system. It is thought that the paucity of functional human B cells and innate immune cells in this model is due to cytotoxic mechanisms mediated by murine innate immune cells and/or the limited reactivity of several mouse cytokines with corresponding human receptors (Hess et al., 2021; Rongvaux et al., 2014, 2013). Ideally, the ultimate goal for studying human aGVHD would be to create mice with a fully humanized adaptive and innate immune system. Over the past decade, great progress has been made in generating mice with a humanized immune system (HIS) via the engraftment of human CD34^+^ HSCs into immunodeficient NSG recipients (Hess et al., 2021; Rongvaux et al., 2014; Martinov et al., 2021). Although new and important information has been reported using these HIS models, development of fully functional innate and adaptive immunity remains limited (Chupp et al., 2024). Recently, Chupp et al. (2024) reported a major breakthrough in HIS development by engrafting immunodeficient, non-irradiated NBSGW and NSGW41 mice (Box 1) with human neonatal cord blood CD34^+^ cells followed by treatment with 17β-estradiol. They found that 17β-estradiol treatment induced a human lymphoid and myeloid immune system that was characterized by marginal zone B cells, germinal center B cells, follicular Th cells and neutrophils, as well as well as formed lymph nodes and intestinal lymphoid tissue (Chupp et al., 2024). In addition, these mice exhibited diverse human B-cell and T-cell antigen receptor repertoires and were able to mount mature T cell-dependent and T cell-independent antibody responses. It will be interesting to assess the immunopathogenesis of aGVHD using this novel HIS model.

## Therapeutic intervention in mouse models of aGVHD

As discussed above, allogeneic mouse models have been invaluable in identifying the role that certain pro-inflammatory cytokines (e.g. IFNγ, IL-1β, IL-2, TNFα and IL-6) and chemokine receptors (e.g. CCR5) play in the pathogenesis of aGVHD. In some cases, some of these inflammatory mediators have been developed for use in clinical studies (Hess et al., 2021; Zeiser and Blazar, 2016, 2017a). Unfortunately, the majority of clinical studies that have evaluated the therapeutic efficacy of immunoneutralizing monoclonal antibodies (mAbs) or selective receptor antagonists for the different inflammatory mediators involved in aGVHD pathogenesis in murine models have shown little or no efficacy in patients (Stolfi et al., 2016; Zeiser and Blazar, 2016; Rayasam and Drobyski, 2021; Enriquez et al., 2020; Blazar et al., 2020; Hess et al., 2021; Kennedy et al., 2021). The reasons for these disappointing results are currently not well understood. It has been suggested that differences in the structure, cell composition, mediators and/or immune responses of the murine versus human immune systems could be responsible for the discouraging therapeutic outcomes. It is known that humans have a more complex lymphatic system that contains larger numbers of lymph nodes than that of mice. The lymph nodes are organized into more complex chains that drain smaller areas of tissue compared to the lymph nodes of mice (Haley, 2003). In addition, the mouse spleen is a major hematopoietic tissue, whereas splenic hematopoietic activity is absent in humans (Haley, 2003; Mestas and Hughes, 2004). In addition to these structural and functional differences, mice and humans exhibit a number of important differences with respect to their innate and adaptive immunity. Mestas and Hughes (2004) provided a detailed overview of these differences, including alterations in the relative abundance of lymphocytes and neutrophils in the systemic circulation, components of B- and T-cell signaling pathways, immunoglobulin subsets, γδ T cells, defensins, cytokines/cytokine receptors, Th1/Th2 differentiation, co-stimulatory molecule expression and function, and chemokine/chemokine receptor expression, to mention just a few. Furthermore, when Shay and coworkers compared the transcriptional profiles of different human and mouse immune cells, they found hundreds of genes with distinctly divergent expression across the different cells (Shay et al., 2013). Using high-throughput sequencing assays on the transcriptome and epigenome of several different tissues obtained from mice and human, Lin et al. (2014) also found extensive RNA expression diversity between the two species. Another feature of aGVHD models that can complicate interpretation of preclinical data generated in allogeneic or xenogeneic mouse models is the fact that human disease is most likely multi-dimensional, involving endless cycles of tissue damage created by alloreactive T-cell activation followed by their infiltration into target tissue, which results in further end-organ damage that promotes the recruitment of a new wave of disease-producing lymphocytes and innate immune cells (Hess et al., 2021). Thus, immunoneutralization or drug blockade of a single mediator may have only a limited effect on overall disease (Gadina et al., 2019; Hess et al., 2021; Schroeder et al., 2018). These concerns have prompted several preclinical studies to assess the efficacy of new therapeutics capable of inhibiting the production of multiple inflammatory mediators.

## Novel therapeutics

### Small molecules

One class of novel small molecules capable of suppressing inflammatory tissue damage in mouse models of aGVHD is selective Janus kinase (JAK) inhibitors (Kim et al., 2024). There are several different JAK family members that, together with signal transducers and activators of transcription (STATs), regulate the transcription of multiple genes upregulated in inflammatory and autoimmune diseases (Kim et al., 2024). JAK1 and JAK2 inhibitors selectively block the downstream signaling of multiple cytokine receptors (Gadina et al., 2019; Hess et al., 2021; Schroeder et al., 2018; Kim et al., 2024; Zeiser et al., 2023). Based upon the exciting preclinical studies that have demonstrated the efficacy of selective JAK inhibitors in mouse models of aGVHD (Courtois et al., 2021; Gadina et al., 2019; Schroeder et al., 2018), new clinical studies have been initiated to test selective JAK inhibitors in patients undergoing allogeneic HSCT or patients with aGVHD (Kim et al., 2024). Ruxolitinib (a JAK1/2 inhibitor) is the first new US Food and Drug Administration (FDA)-approved drug to successfully treat steroid-resistant graft-versus-host (GVHD) in more than 30 years (Zeiser et al., 2020, 2023; Kim et al., 2024).

### Biologics

In addition to JAK inhibitors, a recent clinical study reported the efficacy of a humanized mAb (vedolizumab) to treat lower-gastrointestinal aGVHD (Chen et al., 2024). This study demonstrated in a randomized, double-anonymized, placebo-controlled phase III clinical study that vedolizumab, an mAb specific for T-cell-associated α4β7 integrin, significantly increased the disease-free survival of gastrointestinal aGVHD patients compared to that of the placebo control group (Chen et al., 2024). This clinical trial was based upon data from preclinical mouse models of aGVHD demonstrating that the expression of integrin α4β7 on activated T cells was required for their infiltration and damage to the intestinal mucosa (Dutt et al., 2005; Fu et al., 2019; Petrovic et al., 2004). In these preclinical findings, the reduced extravasation of alloreactive T cells into the gut was associated with markedly reduced production of multiple inflammatory cytokines and chemokines within the tissue, and diminished recruitment and activation of additional inflammatory cells (e.g. monocytes, neutrophils, macrophages) (Fu et al., 2019). Abatacept, another novel biologic currently approved to treat rheumatoid arthritis, psoriatic arthritis and juvenile idiopathic arthritis, has been shown to be effective in preclinical mouse models of aGVHD (Gao et al., 2021; Hess et al., 2021). This cytotoxic T-lymphocyte antigen-4 (CTLA-4) fusion protein suppresses T-cell activation by binding to CD80 and CD86 on DCs, thereby preventing their interaction with T-cell-associated CD28 and second signal activation of these lymphocytes. Similar to JAK inhibitors and vedolizumab, abatacept prevents activation of alloreactive T cells and subsequent generation of inflammatory mediators (Kean et al., 2024). In a recent clinical study, investigators found that when two young children who were undergoing unrelated donor HSCT were treated prophylactically with abatacept in addition to standard GVHD/immunosuppression therapy, survival outcomes were much improved (Kean et al., 2024).

### Cell-based therapeutics

Although exciting, the translational success of these new treatments is limited, as their efficacy is generally short lived, requiring additional/multiple treatments for controlling disease, as with many conventional therapeutics. Thus, investigators have been evaluating novel cell-based therapies that may provide more long-lasting immunosuppressive effects in mouse models of aGVHD. Taylor et al. (2002) were the first to report that infusion of *ex vivo*-activated and expanded murine CD4^+^CD25^+^Foxp3^+^ regulatory T cells (Tregs) (Box 1) suppressed murine aGVHD. Following this landmark study, numerous laboratories have demonstrated that infusion of different populations of Tregs can both prevent and reverse pre-existing disease in standard murine and xenogeneic mouse models of aGVHD (Blazar et al., 2018; Riegel et al., 2020; Zeiser et al., 2023; Dittmar et al., 2024; Hippen et al., 2022). These dramatic preclinical results launched a series of early-phase clinical studies to assess the safety and efficacy of Tregs in limited numbers of patients with preexisting aGVHD (or cGVHD) or prophylactically following allogenic HSCT (Hippen et al., 2022). Overall, investigators have found that infusions of Tregs were well tolerated and provided transient/modest improvement of disease (Hippen et al., 2022). Although these studies are quite promising, randomized and placebo-controlled clinical studies will be needed to demonstrate the efficacy and reproducibility of these Treg protocols.

Another population of immunosuppressive cells that are being evaluated for their ability to attenuate aGVHD in mice and humans is the mesenchymal stromal cells (MSCs), which are present in BM, blood, umbilical blood, adipose tissue and placenta (Kadri et al., 2023; Zeiser et al., 2023; Wuttisarnwattana et al., 2023). Upon activation by inflammatory cytokines (such as IFNγ, IL-1, IL-6 and TNFα), MSCs produce immunosuppressive cytokines, such as IL-10, transforming growth factor-β, hepatocyte growth factor, prostaglandin E2, nitric oxide and indoleamine 2,3-dioxygenase (Glenn and Whartenby, 2014; Le Blanc and Mougiakakos, 2012). Although a number of preclinical studies have demonstrated MSC-mediated suppression of aGVHD, others have not (Kadri et al., 2023). Currently, the specific mechanisms responsible for immune suppression *in vivo*, as well as the location(s) of cell engraftment, have not been definitively identified. Nevertheless, a number of early-phase clinical studies are underway to assess the efficacy of MSCs in suppressing aGVHD (Kadri et al., 2023; Zeiser et al., 2023). As observed in murine models, some, but not all, of the MSC clinical studies have demonstrated significant disease improvement (Kadri et al., 2023). In addition, several other immunosuppressive cell populations have been suggested as candidates for treating aGVHD, including myeloid-derived suppressor cells (Feng et al., 2023), regulatory DCs (Scroggins and Schlueter, 2023), invariant NK cells (Du et al., 2017) and regulatory B cells (Rosser and Mauri, 2015; Rosser and Mauri, 2021).

Despite the wealth of information generated from mouse models and the success of translating a few of the more recent preclinical data into new and effective treatments of human aGVHD, bench-to-bedside translation has remained relatively low (Stolfi et al., 2016; Zeiser and Blazar, 2016; Rayasam and Drobyski, 2021; Enriquez et al., 2020; Blazar et al., 2020; Hess et al., 2021). Accumulating preclinical data suggest that these disappointing outcomes are related to differences in the protocols used in murine models of aGVHD compared to those used in clinical allogeneic HSCT (Table 3) (Blazar et al., 2020; Enriquez et al., 2020; Stolfi et al., 2016; Enriquez et al., 2023; Zeiser and Blazar, 2016). In the next section, we discuss how these differences may affect translation of preclinical data to patient treatment and provide suggestions as to how this information could be used to increase the translatability of mouse models of aGVHD.

**Table 3.** Major differences between mouse models and clinical protocols of allogeneic hematopoietic stem cell transplantation*

Characteristics	Mice	Humans
Genotype	Inbred; genetically identical	Outbred; heterozygous
Age	Homogeneous; very young; 8-12 weeks	Heterogeneous: young, adolescent, middle age, older age in some cases
Environment	Specific pathogen-free conditions; suboptimal housing temperatures	Much greater microbial diversity; extensive exposure to pathogens and infectious agents; experience marked fluctuations in ambient temperature
Phenotype	Lean, healthy and young	Highly variable, with increasing numbers of young-adult and middle-aged patients with obesity; medically infirm individuals
Conditioning protocol	Lethal total-body radiation	Chemotherapy±lethal radiation; reduced intensity conditioning
Donor cells	Hematopoietic stem cells isolated from bone marrow; additional donor T cells are required for aGVHD	CD34^+^ progenitor cells isolated from bone marrow, G-CSF-treated blood or umbilical cord blood

*Table compiled from data presented in Blazar et al. (2020); Schroeder and DiPersio (2011); Stolfi et al. (2016); Zeiser and Blazar (2016). aGVHD, acute graft-versus-host disease; G-CSF, granulocyte colony-stimulating factor.

## Factors limiting bench-to-bedside translation using mouse models

### Pretransplant conditioning protocols

#### Chemotherapy, TBI and immunosuppressive medications

As discussed previously, patients that undergo allogeneic HSCT receive a pretransplant conditioning regimen that includes high-dose chemotherapy (busulfan, fludarabine, cyclophosphamide) and/or TBI, which are effective in eradicating malignant cells and preparing the bone marrow for HSC engraftment. In addition, prophylactic administration of one or more immunosuppressive medications (e.g. tacrolimus, methotrexate, anti-thymocyte globulin) is used to suppress alloreactive immune responses, promote engraftment of the transplanted cells and reduce the development of aGVHD (Shaffer et al., 2024). New clinical studies have reported that post-transplant administration of cyclophosphamide reduces the development of aGVHD in recipients who received HLA-disparate HSCs (Shaffer et al., 2024; Shaw et al., 2021; Bolanos-Meade et al., 2023). Of note, these clinical studies were based on the important preclinical findings that post-transplant administration of cyclophosphamide limited the development of aGVHD in mouse models of allogeneic HSCT (O'Donnell and Jones, 2023). Unfortunately, these types of preclinical studies using clinically relevant treatment protocols to model allogenic HSCT in mice are very rare. The paucity of these types of studies could limit the translatability of mouse models of disease.

#### Intestinal tissue damage: cause or consequence of aGVHD?

As noted previously, conventional pretransplant conditioning protocols in mice (e.g. TBI) damage a variety of different tissues, producing numerous inflammatory cytokines and mediators that promote the activation and recruitment of alloantigen-specific T cells to different target tissues (Khuat et al., 2021; Hill and Koyama, 2020; Blazar et al., 2020; Zeiser et al., 2016; Castor et al., 2012; Mapara et al., 2006; Chewning et al., 2013; Dudakov et al., 2017; Schroeder and DiPersio, 2011; Shono et al., 2014, 2010; Stolfi et al., 2016; Zeng et al., 2017; Hill et al., 2021). Several preclinical and clinical studies suggest that the severity of aGVHD is directly related to the magnitude of intestinal injury produced by pretransplant conditioning protocols (Nalle et al., 2014; Nalle and Turner, 2015; Zeiser and Blazar, 2017a; Hill et al., 1997, 2021; Johansson and Ekman, 2007; Hill and Ferrara, 2000). These correlative data suggest that damage to the gut by myeloablative conditioning promotes the translocation of intestinal bacteria and their products into the gut interstitium, mesenteric lymph nodes and systemic circulation, where they initiate alloreactive T cell-mediated aGVHD.

An important question that has been debated over the past few years is whether gut damage is a cause or consequence of multi-organ aGVHD (Nalle and Turner, 2015). In an attempt to differentiate between these two possibilities, Nalle et al. (2014) injected unfractionated, alloreactive splenocytes into NK cell-depleted mice in which recombination activating gene-1 (*Rag1*) was inactivated (NK^−^/*Rag1*^−/−^) to determine whether they would develop aGVHD (Table 1). Notably, these immunodeficient mice were engrafted with allogeneic T cells without the use of toxic pretransplant conditioning. This study demonstrated that intestinal damage is not required for development of aGVHD when MHC-mismatched splenocytes are engrafted. However, gut injury was required when minor histocompatibility antigen (miHA; Box 1)-mismatched (Box 1) splenocytes were used (Nalle et al., 2014). A major advantage of this model is that it creates immunodeficient mice without the use of tissue-damaging conditioning protocols. One potential limitation with the NK^−^/*Rag1*^−/−^ model is whether the pathogenetic immune responses are similar to those described in the more conventional mouse models that use TBI as the conditioning protocol.

Because unfractionated splenocytes were used as a source for disease-producing effector cells in this model, the specific T-cell populations responsible for inducing aGVHD were not determined. To address this question, our group investigated whether allogeneic CD4^+^ T cells alone could induce aGVHD when injected into immunodeficient NK^−^/*Rag1*^−/−^ mice or mice devoid of T, B and NK cells by virtue of deletions of their recombination activating gene-2 (*Rag2*; Box 1) gene and interleukin-2 receptor common γ (*Il2rg*) gene (McDaniel Mims et al., 2019). We found that adoptive transfer of naïve CD4^+^CD62L^+^CD25^−^ T cells from allogeneic donors into either mouse model induced clinical and histological features of aGVHD, including weight loss, inflammatory cytokine production and tissue inflammation (McDaniel Mims et al., 2019). In addition, engraftment of these alloantigen-specific T cells induced inflammation of the liver and skin, as well as severe anemia due to remarkable BM and spleen damage (McDaniel Mims et al., 2019). Interestingly, these mice did not develop histological evidence of inflammatory tissue damage to the gut or lungs. These findings demonstrated that CD4^+^ T cells are both necessary and sufficient to induce aGVHD in lymphopenic recipients in the absence of toxic pretransplant conditioning and confirm the studies of Nalle et al. (2014) demonstrating that intestinal damage is not required for the development of aGVHD in this model. The findings described above are similar to those reported using mouse models of xGVHD in which injection of human BM mononuclear cells into unconditioned, immunodeficient NBSGW mice induced aGVHD in the absence of tissue damage (Hess et al., 2021, 2020). Based upon these data, Hess et al. (2021) suggested that although cytotoxic inflammatory mediators are not required for initiation of aGVHD, they most likely influence the progression, prevalence and severity of disease.

**Fig. 3.**
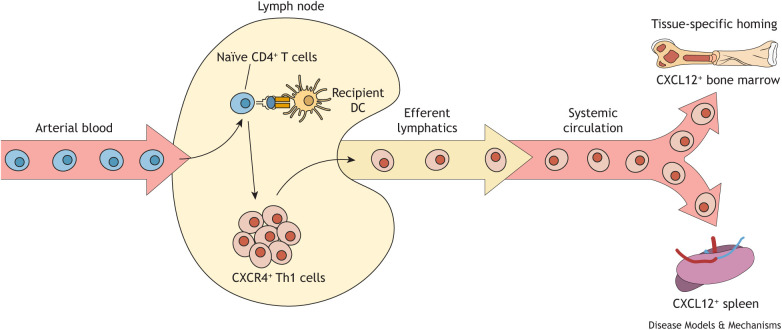
**Alloreactive Th1 cells selectively traffic to and damage the bone marrow and spleen of mice subjected to reduced intensity conditioning.** Intravenous administration of naïve allogeneic CD4^+^ T cells into recipients subjected to reduced intensity conditioning (sublethal irradiation) induces bone marrow failure and spleen damage but no injury to any other tissue. The interaction of allogeneic T cells with recipient lymph node-residing dendritic cells (DCs) induces the activation, polarization and expansion of Th1 effector cells that express the chemokine receptor CXCR4. These alloreactive effector cells leave the lymph nodes via the efferent lymphatics and enter the systemic circulation, in which they selectively traffic to the bone marrow and spleen, which express CXCL12, the specific ligand for CXCR4.

Historically, the use of myeloablative conditioning has been reserved for relatively young patients because its use in older, medically infirm individuals increases morbidity and mortality (Gyurkocza and Sandmaier, 2014; Shimoni et al., 2016; Stolfi et al., 2016). These clinical observations prompted the development of reduced intensity conditioning (RIC) protocols to increase safety, reduce disease relapse and increase overall survival, thereby expanding the availability of HSCT (Gyurkocza and Sandmaier, 2014; Pingali and Champlin, 2015). We recently reported how RIC prior to engraftment of allogeneic T cells affects the onset, severity and location of aGVHD (Enriquez et al., 2023). Using a modification of a mouse model described by Chen et al. (2004) (Table 1), we found that intravenous administration of small numbers of flow-sorted CD4^+^CD25^−^ conventional T cells (Box 1) from allogeneic donors into recipients subjected to RIC (sublethal irradiation) induced severe wasting disease characterized by anemia and cytopenia (Box 1) that was associated with extensive BM failure and spleen damage (Enriquez et al., 2023). We did not observe damage to those tissues (gut, liver, skin) usually targeted in conventional, lethally irradiated mouse models of aGVHD. Adoptive transfer of allogeneic T cells into RIC-treated recipients also induced striking dysbiosis (Box 1), characterized by marked reductions in commensal/beneficial bacteria that are known to produce lactate and short-chain fatty acids (SCFAs) (Enriquez et al., 2023)*.* It should be noted that lactate and certain SCFAs (e.g. butyrate) are known to promote hematopoiesis, and thus their loss can negatively affect the production of new blood cells (Lee et al., 2021). Variations of this RIC model have also been used to model immune-mediated aplastic anemia via adoptive transfer of unfractionated, allogeneic lymph node or spleen cells into sublethally irradiated recipients (Arieta Kuksin et al., 2015; Chen et al., 2015b). These studies, as well as findings from clinical studies, suggest that hematopoietic tissues, such as the BM and spleen, are particularly sensitive to alloreactive T cell-mediated damage (Chen et al., 2015a; Chewning et al., 2013; McDaniel Mims et al., 2019; Muskens et al., 2021; Shono et al., 2014, 2010; Zeiser and Teshima, 2021). Indeed, alloreactive T cells appear to selectively traffic to and damage BM and spleen in RIC-treated mice (Fig. 3). Taken together, these findings suggest that although RIC dramatically reduces tissue damage in non-lymphoid tissue, hematopoietic tissue injury can persist, rendering mice (and possibly RIC-treated patients) susceptible to recurrent infections and bacteremia (Box 1).

#### Effects on the intestinal microbiota

It is well known that, in addition to promoting microbial translocation, myeloablative conditioning protocols can dramatically alter intestinal bacterial populations prior to HSCT. Alterations in gut bacteria (called dysbiosis) produced by these conditioning protocols include marked loss of diversity and abundance of anaerobic bacteria (e.g. Clostridia), whereas the abundance of potential pathogens (e.g. Enterococci, Streptococci and/or Escherichia) is increased (Fredricks, 2019; Peled et al., 2020; Shono et al., 2016; Staffas et al., 2017; van Lier et al., 2023). These data prompted investigators to assess the protective effects of antibiotic administration prior to and/or following pretransplant conditioning. Some studies have reported that antibiotic treatment of mice or humans undergoing HSCT attenuates the onset and/or severity of aGVHD (Fredricks, 2019; Schwab et al., 2014), whereas other studies reported that administration of certain antibiotics can exacerbate aGVHD and increase mortality by damaging bacterial communities that are critical for maintaining intestinal epithelial cell viability and mucosal barrier function (Fredricks, 2019; McDaniel Mims et al., 2021; van Lier et al., 2023). We found that oral administration of a broad-spectrum antibiotic cocktail for 7 days prior to and 4 weeks following engraftment of allogeneic CD4^+^ T cells into non-conditioned, immunodeficient (e.g. NK^−^/*Rag1*^−/−^) mice exacerbated aGVHD-induced BM and spleen damage compared to that in their untreated controls engrafted with allogeneic T cells (McDaniel Mims et al., 2021). Tissue damage was associated with severe anemia and a remarkable reduction in fecal bacterial load and diversity (McDaniel Mims et al., 2021). Our data agree with clinical findings suggesting that prolonged pre- and post-transplant administration of high-dose, broad-spectrum antibiotics in patients undergoing allogeneic HSCT is a major contributor to microbial damage and severe dysbiosis (Fredricks, 2019; Staffas et al., 2017; van Lier et al., 2023). Indeed, these observations have important hematologic implications given that microbe-associated molecular patterns (e.g. lipopolysaccharide, peptidoglycan, flagellin), as well as certain bacterial-derived products (SCFAs, lipopolysaccharide, lactate, etc.), interact with HSCs and hematopoietic progenitor cells (HSPCs) to induce their proliferation and differentiation into all of the cellular elements of blood (Josefsdottir et al., 2017; Lee et al., 2021).

#### Novel approaches for pretransplant conditioning

Recent ground-breaking, preclinical studies have reported the efficacy of a novel pretransplant conditioning protocol that uses an anti-mouse CD45 antibody-drug conjugate (ADC) that is coupled to the cytotoxic DNA crosslinker tesirine (Saha et al., 2022, 2024). CD45-ADC binds directly to all BM immune cells, HSCs and HSPCs that express CD45 on their surface, thereby initiating its internalization and cell death, as free tesirine is toxic to these cells. When unconditioned C57BL/6 (H-2^b^) (Box 1) mice were treated with a single dose of CD45-ADC, they exhibited a rapid and marked loss of BM HSCs and HSPCs within 3 days post-treatment (Saha et al., 2022). This response could be partially rescued by the engraftment of ADC-treated Bl6 recipients with fully MHC-mismatched BM cells from CByJ.SJL(B6)-*Ptprc^a^*/J CD45.1 (H-2^d^) mice (Box 1), resulting in 95% donor chimerism (Box 1) in peripheral blood, with donor myeloid cells, B cells and T cells achieving 95% long-term engraftment (Saha et al., 2022). In a recent follow-up study, these authors compared the development of aGVHD and survival in CD45-ADC-conditioned C57BL/6 mice that received allogeneic BM cells from BALB/c (H-2^d^) mice (Box 1) to C57BL/6 mice that were conditioned with lethal TBI and then engrafted with BALB/c BM cells (Saha et al., 2024). They found that 88% of CD45-ADC-conditioned mice (Box 1) survived for more than 18 weeks post-transplant, whereas only 10% of TBI-conditioned recipients survived the same time following transplantation (Saha et al., 2024). Importantly, clinical GVHD scores in CD45-ADC-conditioned mice were significantly reduced compared to those in their TBI-conditioned counterparts (Saha et al., 2024). Similar results were observed when donors and recipients were reversed (C57BL/6→BALB/c). Using these same protocols, Saha et al. (2024) then determined whether CD45-ADC conditioning followed by allogeneic HSCT would prove effective in treating three different mouse models of Fanconi anemia (Box 1). Fanconi anemia is an inherited genetic disease characterized by BM failure, anemia, pancytopenia, myelodysplasia and leukemia, for which HSCT is the only available cure (Saha et al., 2024). Saha et al. (2024) found that CD45-ADC conditioning followed by transplantation of fully allogeneic BM significantly increased survival and reduced aGVHD compared to that in Fanconi anemia mice that received lethal TBI and allogeneic HSCT. In addition, these investigators reported that administration of anti-human CD45-ADC to Rhesus monkeys (Box 1) produced robust myeloablation that was similar to that produced by lethal TBI (Saha et al., 2024). Taken together, these data demonstrate that CD45-ADC conditioning enhances engraftment and hematopoiesis following allogeneic HSCT while significantly reducing aGVHD. Results from these studies are particularly exciting as future preclinical HSCT investigations using this novel conditioning protocol could reveal new strategies to protect recipients from alloreactive T cells.

## Animal housing environment

Another factor that is likely to limit bench-to-bedside translation in mouse models is their housing environment. Specific pathogen-free (SPF) animal care facilities are designed to exclude pathogenic viruses, bacteria and parasites by using multiple ‘barriers’ and restricted access of staff, as well as by employing high-efficiency particulate air (HEPA) filters and sterilized drinking water for all cages. These precautions create a uniform ultra-hygienic environment that provides more reproducible immune responses and phenotypes in the animals housed in this facility. Despite the use of these ultra-hygienic conditions, the phenotypes of different mouse models of inflammatory diseases may be markedly different from one institution to another (Enriquez et al., 2020; Ericsson et al., 2015; Ivanov et al., 2009; Kriegel et al., 2011; Webb et al., 2018). This could be due to differences in animal vendor, diet and/or water treatment, any one of which may markedly alter the gut microbiota of mice (Ericsson et al., 2015; Kriegel et al., 2011; Webb et al., 2018). Major differences in the incidence and severity of chronic gut inflammation in mouse models of inflammatory bowel disease have been shown to occur following relocation of mice to a different animal care facility or change of vendor (Ericsson et al., 2015; Fox et al., 2011; Webb et al., 2018; Stepankova et al., 2007; Yang et al., 2013). An example of how housing conditions can affect the onset and/or severity of aGVHD, Li et al. (2020) recently reported that injection of unfractionated, allogeneic C57BL/6 lymph node cells into CByB6F1 recipients housed under non-SPF conditions [e.g. conventional housing conditions (Box 1)] induced significantly less allogeneic T-cell expansion and BM damage compared to that in mice housed under SPF conditions. These responses were associated with greater microbial diversity within mice housed under convention housing conditions compared to that in SPF-housed recipients (Li et al., 2020). These findings illustrate how differences in the composition of intestinal microbiota in mice housed under different conditions within the same animal facility may alter the onset and/or severity of inflammation, thereby affecting the reproducibility of these models. In contrast to the ‘ultra-clean’ SPF environment, humans are continuously exposed to a spectrum of different pathogenic and nonpathogenic microorganisms that both protect us and shape our immune system to fight future infectious microbes (Enriquez et al., 2020). Investigators have reported that mice housed under SPF conditions have remarkably underdeveloped immune systems compared to those of ‘dirty’ mice housed in pet stores or residing in the wild [i.e. feral mice (Box 1)] (Beura et al., 2016; Liu et al., 2024; Rehermann et al., 2024). Studies have shown that mice need to be exposed to a diverse array of naturally occurring microorganisms to develop a mature immune system that more closely resembles that of humans (Beura et al., 2016; Masopust et al., 2017; Rosshart et al., 2017; Burger et al., 2023; Liu et al., 2024). And, as discussed below, feral mice possess a diverse intestinal microbiota that promotes the development of a fully functional immune system, protecting them from numerous and potentially lethal infectious agents, carcinogens and inflammatory mediators.

There are now numerous examples of the translational importance of microbiota for the modeling of human infectious and inflammatory diseases. For example, Beura et al. (2016) reported that when pet store mice or SPF mice co-housed with pet store mice were infected with *Listeria monocytogenes* (Box 1), their bacterial load was reduced by more than 10,000-fold compared to that in standard SPF animals. Another study reported that colonic inflammation and numbers of invasive adenomas (Box 1) and adenocarcinomas (Box 1) were significantly reduced in feral microbiota-colonized mice relative to SPF-colonized controls in a mouse model of colorectal cancer (Rosshart et al., 2017). The National Institute of Allergy and Infectious Diseases recently convened a 2-day workshop to discuss how exposure of mice to diverse microbial communities may improve our understanding of human immune system development and disease (Liu et al., 2024). Participants, including several of the investigators referenced above, presented data related to the development and use of microbial-experienced mouse models of infectious and inflammatory diseases. In addition, the consortium discussed the challenges and possible solutions when using ‘dirty’ mice to model human immunity. A recent ground-breaking study by Lo et al. (2024) reported how the use of laboratory mice colonized with feral microbiota could advance our understanding of the immunopathogenesis of immune-related adverse events induced by anti-tumor biologics called immune checkpoint inhibitors. Administration of checkpoint inhibitors such as anti-CTLA-4 (Box 1) mAb induces severe colitis in a subset of cancer patients undergoing treatment with this immunotherapeutic antibody (Waldman et al., 2020). Unfortunately, checkpoint inhibitors fail to induce colitis in mice housed under SPF conditions. Lo et al. (2024) reported that treating feral microbiota-colonized mice with anti-CTLA-4 antibodies induced robust colitis, demonstrating the feasibility of using feral microbiota as models of inflammatory diseases.

Another factor that can affect the translatability of mouse models of disease is housing temperature. All animal care facilities in the USA house mice at a standard temperature (ST) range of 22-24°C. This temperature is below the murine thermoneutral temperature (TNT) range of 30-32°C (Bucsek et al., 2018; Hylander et al., 2019; Hylander and Repasky, 2016; Karp, 2012). It is well known that ST housing creates mild, but chronic, cold stress that can alter a number of different immune responses compared with those in mice housed at their TNT (Enriquez et al., 2020; Bucsek et al., 2018; Hylander et al., 2019, 2023; Hylander and Repasky, 2016; Karp, 2012; James et al., 2023). Both primary and secondary lymphoid tissue are innervated by the sympathetic nervous system, which can be activated by ST-induced cold stress, resulting in the release of norepinephrine, which modulates a number of different immune cell responses in a receptor/ligand-specific manner (Hylander et al., 2023; James et al., 2023). ST-induced, norepinephrine-mediated alterations in disease phenotypes have also been reported in mouse aGVHD, cancer and cardiovascular disease models (Hylander et al., 2023; James et al., 2023). Furthermore, certain gut microbial communities are significantly altered in mice housed at ST versus TNT (Hylander et al., 2023). Indeed, changes in environmental temperature may exert profound effects on intestinal homeostasis and microbial composition in mice (Chevalier et al., 2015; Hylander et al., 2019, 2023; Karl et al., 2018). An interesting observation, reported by numerous investigators using the conventional, lethally irradiated mouse model of aGVHD, is that engraftment of allogeneic BM alone into recipients subjected to lethal irradiation and housed at ST does not induce aGVHD unless the BM is supplemented with a source of allogeneic T cells (e.g. splenocytes and/or isolated T cells) (Hill and Koyama, 2020; Schroeder and DiPersio, 2011; Stolfi et al., 2016; Zeiser and Blazar, 2017a). This contrasts markedly with human HSCT protocols, which attempt to remove all allogeneic T cells prior to HSC administration. Leigh et al. (2015) reported that lethally irradiated mice housed at TNT and engrafted with BM alone did in fact develop aGVHD, suggesting that ST-mediated cold stress was suppressing the development of inflammatory tissue damage. Furthermore, they found that disease suppression at ST was mediated by the interaction between norepinephrine and the β2-adrenergic receptor (Leigh et al., 2015). Because most aGVHD mouse models use lethal TBI as the pretransplant conditioning protocol and ST housing, we investigated whether housing temperature affects the onset and severity of aGVHD in the RIC mouse model of T cell-mediated BM and spleen damage described above. In contrast to the study reported by Leigh et al. (2015), which used a conventional model of aGVHD, we found that the survival of allogeneic T cell-engrafted mice housed at ST to be significantly reduced compared to that of their counterparts housed at TNT (Enriquez et al., 2023). We are currently investigating the mechanisms by which ST decreases survival in this model. Together, these findings suggest that relatively modest differences in housing temperature may produce major changes in disease phenotype.

## Inbred versus outbred mice

A report recently estimated that >5 million mice are used every year according to annual use reports for rats and mice from 16 of the top 30 National Institutes of Health (NIH)-funded US research institutions. The author extrapolated this number to all US institutions, estimating that >100 million mice are used in research in the USA annually (Carbone, 2021). Others have argued that this is an overestimation and that annual mouse use lies somewhere between 25 and 40 million/year in the USA (Grimm, 2021). Despite these different estimates, it is fair to say that large numbers of mice are used for biomedical research. Although Carbone (2021) could not provide a breakdown of the inbred versus outbred mice used by the different institutions, it is highly likely that inbred mice are used for the large majority of preclinical studies investigating the immunopathogenesis of inflammatory diseases. Indeed, all mouse models of aGVHD use young, lean and healthy inbred strains of mice as surrogates for genetically diverse humans (Table 3). In contrast, patients undergoing HSCT are likely to be medically infirm, older and/or obese (Blazar et al., 2020; Stolfi et al., 2016). Preclinical studies have shown that altering one or more of these risk factors could have a major impact on disease phenotypes in mouse models (Blazar et al., 2020; Mirsoian et al., 2014). Although the use of inbred mice has been critical to our understanding of immunology and immunopathology, it is not clear whether the immune responses of these genetically constrained animals recapitulate the diverse immune responses found in humans (Enriquez et al., 2020). It is known that human T-cell responses are more heterogeneous than those of inbred mice. In addition, specific immune responses and disease phenotypes can be quite different in inbred versus outbred mice (Graham et al., 2017, 2015; Martin et al., 2017; Nikodemova and Watters, 2011; Reichenbach et al., 2013). Thus, the exclusive use of healthy inbred mice could limit the bench-to-beside translation of data obtained from aGVHD studies (Enriquez et al., 2020; Blazar et al., 2020; Stolfi et al., 2016).

To our knowledge, no studies have used outbred mice to study the immunopathogenesis of aGVHD. Therefore, our group engrafted RIC-treated outbred CD1 mice with inbred (or outbred) allogeneic CD4^+^ T cells and then assessed recipient mice housed at ST or TNT for tissue damage. We found that engraftment of large numbers of allogeneic T cells into RIC-treated CD1 mice did not induce aGVHD in any tissue in mice housed at ST or TNT. These data suggest that outbred mice subjected to RIC are resistant to T cell-mediated aGVHD in this and possibly other models of aGVHD (Enriquez et al., 2023). Because MHC II disparity alone was incapable of driving T cell-mediated tissue damage in these outbred mice, it is reasonable to suggest that uncharacterized, regulatory immune mechanisms contribute to disease resistance. Other investigators have reported similar results when attempting to induce autoimmune encephalomyelitis or diabetes in outbred recipients (Marin et al., 2014; Maron et al., 1999). Although we did not determine the mechanisms by which the outbred CD1 mice remained disease free in our study, we did observe significantly larger numbers of BM- and spleen-residing B cells and myeloid cells relative to those in inbred mice that developed aGVHD (Enriquez et al., 2023). These data, together with those reported by Marin et al. (2014) for their mouse model of autoimmune encephalitis, suggest that within the B-cell and/or myeloid cell populations present in T cell-engrafted CD1 mice reside substantial numbers of immunosuppressive regulatory B cells and/or myeloid derived suppressor cells (Enriquez et al., 2023). It will be interesting to see whether future studies can identify the mechanisms responsible for suppression of aGVHD in outbred mice.

Although outbred mice have been used extensively in toxicological, pharmacological and infectious disease research, their use in modeling inflammatory diseases has been limited (Enriquez et al., 2020; Chia et al., 2005). In addition, the genotypes of commercially available stocks of outbred mice are either unavailable or poorly defined (Chia et al., 2005). These concerns have led investigators to generate genetically diverse reference populations of mice that are referred to as Collaborative Cross mice (Collaborative Cross Consortium, 2012; Threadgill and Churchill, 2012). These highly outbred mice were generated by combinational breeding schemes using eight unique and genetically diverse founder strains of mice that included three inbred strains, two inbred mouse models of diseases (type 1 diabetes and obese/type 2 diabetes mice) and three wild-derived strains (Graham et al., 2017; Leist and Baric, 2018; Phillippi et al., 2014; Threadgill and Churchill, 2012). Using multi-combinational and sibling breeding protocols, more than 60 Collaborative Cross recombinant inbred strains have been generated. Each mouse within a specific Collaborative Cross recombinant inbred strain is genetically identical. It will be interesting to determine how these mice respond to allogeneic HSCT protocols.

## Concluding remarks

The use of mouse models to identify new and more effective therapeutic strategies for the treatment of inflammatory diseases is a mainstay for preclinical studies. Unfortunately, the bench-to-bedside transition of promising therapeutic strategies observed in mouse models of aGVHD has been disappointing. The reasons for the limited translation of preclinical data have not been completely defined; however, several concerns associated with murine models have raised important questions regarding the translational nature of using lethal TBI as a conditioning protocol for inbred mice housed under ultra-hygienic and stress-inducing temperatures. Indeed, these conditioning and housing protocols, as well as the use of inbred strains of mice, markedly alter murine microbiota, their immune responses and the immunopathogenesis of aGVHD. Designing studies that more accurately reflect the human environment and treatment protocols are likely to increase the translatability of data obtained from preclinical studies.

## References

[DMM052084C1] Arieta Kuksin, C., Gonzalez-Perez, G. and Minter, L. M. (2015). CXCR4 expression on pathogenic T cells facilitates their bone marrow infiltration in a mouse model of aplastic anemia. *Blood.* 125, 2087-2094. 10.1182/blood-2014-08-594796 25647836 PMC4375106

[DMM052084C2] Baumeister, S. H. C., Rambaldi, B., Shapiro, R. M. and Romee, R. (2020). Key aspects of the immunobiology of haploidentical hematopoietic cell transplantation. *Front. Immunol.* 11, 191. 10.3389/fimmu.2020.00191 32117310 PMC7033970

[DMM052084C3] Beura, L. K., Hamilton, S. E., Bi, K., Schenkel, J. M., Odumade, O. A., Casey, K. A., Thompson, E. A., Fraser, K. A., Rosato, P. C., Filali-Mouhim, A. et al. (2016). Normalizing the environment recapitulates adult human immune traits in laboratory mice. *Nature* 532, 512-516. 10.1038/nature17655 27096360 PMC4871315

[DMM052084C153] Blaser, B. W., Roychowdhury, S., Kim, D. J., Schwind, N. R., Bhatt, D., Yuan, W., Kusewitt, D. F., Ferketich, A. K., Caligiuri, M. A. and Guimond, M. (2005). Donor-derived IL-15 is critical for acute allogeneic graft-versus-host disease. *Blood* 105, 894-901. 10.1182/blood-2004-05-1687 15374888

[DMM052084C154] Blazar, B. R., Carroll, S. F. and Vallera, D. A. (1991). Prevention of murine graft-versus-host disease and bone marrow alloengraftment across the major histocompatibility barrier after donor graft preincubation with anti-LFA1 immunotoxin. *Blood* 78, 3093-3102. 1954395

[DMM052084C4] Blazar, B. R., Murphy, W. J. and Abedi, M. (2012). Advances in graft-versus-host disease biology and therapy. *Nat. Rev. Immunol.* 12, 443-458. 10.1038/nri3212 22576252 PMC3552454

[DMM052084C5] Blazar, B. R., MacDonald, K. P. A. and Hill, G. R. (2018). Immune regulatory cell infusion for graft-versus-host disease prevention and therapy. *Blood* 131, 2651-2660. 10.1182/blood-2017-11-785865 29728401 PMC6032895

[DMM052084C6] Blazar, B. R., Hill, G. R. and Murphy, W. J. (2020). Dissecting the biology of allogeneic HSCT to enhance the GvT effect whilst minimizing GvHD. *Nat. Rev. Clin. Oncol.* 17, 475-492. 10.1038/s41571-020-0356-4 32313224 PMC7901860

[DMM052084C7] Bolanos-Meade, J., Hamadani, M., Wu, J., Al Malki, M. M., Martens, M. J., Runaas, L., Elmariah, H., Rezvani, A. R., Gooptu, M., Larkin, K. T. et al. (2023). Post-transplantation cyclophosphamide-based graft-versus-host disease prophylaxis. *N. Engl. J. Med.* 388, 2338-2348. 10.1056/NEJMoa2215943 37342922 PMC10575613

[DMM052084C8] Brehm, M. A., Kenney, L. L., Wiles, M. V., Low, B. E., Tisch, R. M., Burzenski, L., Mueller, C., Greiner, D. L. and Shultz, L. D. (2019). Lack of acute xenogeneic graft- versus-host disease, but retention of T-cell function following engraftment of human peripheral blood mononuclear cells in NSG mice deficient in MHC class I and II expression. *FASEB J.* 33, 3137-3151. 10.1096/fj.201800636R 30383447 PMC6404556

[DMM052084C155] Bruck, F., Belle, L., Lechanteur, C., de Leval, L., Hannon, M., Dubois, S., Castermans, E., Humblet-Baron, S., Rahmouni, S., Beguin, Y. et al. (2013). Impact of bone marrow-derived mesenchymal stromal cells on experimental xenogeneic graft-versus-host disease. *Cytotherapy* 15, 267-279. 10.1016/j.jcyt.2012.09.003 23265769

[DMM052084C9] Bucsek, M. J., Giridharan, T., MacDonald, C. R., Hylander, B. L. and Repasky, E. A. (2018). An overview of the role of sympathetic regulation of immune responses in infectious disease and autoimmunity. *Int. J. Hyperthermia* 34, 135-143. 10.1080/02656736.2017.1411621 29498310 PMC6309867

[DMM052084C10] Burger, S., Stenger, T., Pierson, M., Sridhar, A., Huggins, M. A., Kucaba, T. A., Griffith, T. S., Hamilton, S. E. and Schuldt, N. J. (2023). Natural Microbial Exposure from the Earliest Natural Time Point Enhances Immune Development by Expanding Immune Cell Progenitors and Mature Immune Cells. *J. Immunol.* 210, 1740-1751. 10.4049/jimmunol.2300061 37074206 PMC10192123

[DMM052084C11] Carbone, L. (2021). Estimating mouse and rat use in American laboratories by extrapolation from Animal Welfare Act-regulated species. *Sci. Rep.* 11, 493. 10.1038/s41598-020-79961-0 33436799 PMC7803966

[DMM052084C12] Carlson, M. J., West, M. L., Coghill, J. M., Panoskaltsis-Mortari, A., Blazar, B. R. and Serody, J. S. (2009). In vitro-differentiated TH17 cells mediate lethal acute graft-versus-host disease with severe cutaneous and pulmonary pathologic manifestations. *Blood* 113, 1365-1374. 10.1182/blood-2008-06-162420 18957685 PMC2637199

[DMM052084C13] Castor, M. G., Pinho, V. and Teixeira, M. M. (2012). The role of chemokines in mediating graft versus host disease: opportunities for novel therapeutics. *Front. Pharmacol.* 3, 23. 10.3389/fphar.2012.00023 22375119 PMC3285883

[DMM052084C14] Chen, J., Lipovsky, K., Ellison, F. M., Calado, R. T. and Young, N. S. (2004). Bystander destruction of hematopoietic progenitor and stem cells in a mouse model of infusion-induced bone marrow failure. *Blood* 104, 1671-1678. 10.1182/blood-2004-03-1115 15166031

[DMM052084C15] Chen, J., Desierto, M. J., Feng, X., Biancotto, A. and Young, N. S. (2015a). Immune-mediated bone marrow failure in C57BL/6 mice. *Exp. Hematol.* 43, 256-267. 10.1016/j.exphem.2014.12.006 25555453 PMC4378590

[DMM052084C16] Chen, J., Feng, X., Desierto, M. J., Keyvanfar, K. and Young, N. S. (2015b). IFN-gamma-mediated hematopoietic cell destruction in murine models of immune-mediated bone marrow failure. *Blood* 126, 2621-2631. 10.1182/blood-2015-06-652453 26491068 PMC4671109

[DMM052084C17] Chen, Y. B., Mohty, M., Zeiser, R., Teshima, T., Jamy, O., Maertens, J., Purtill, D., Chen, J., Cao, H., Rossiter, G. et al. (2024). Vedolizumab for the prevention of intestinal acute GVHD after allogeneic hematopoietic stem cell transplantation: a randomized phase 3 trial. *Nat. Med.* 30, 2277-2287. 10.1038/s41591-024-03016-4 38844797 PMC11333288

[DMM052084C18] Chevalier, C., Stojanovic, O., Colin, D. J., Suarez-Zamorano, N., Tarallo, V., Veyrat-Durebex, C., Rigo, D., Fabbiano, S., Stevanovic, A., Hagemann, S. et al. (2015). Gut microbiota orchestrates energy homeostasis during cold. *Cell* 163, 1360-1374. 10.1016/j.cell.2015.11.004 26638070

[DMM052084C19] Chewning, J. H., Zhang, W., Randolph, D. A., Swindle, C. S., Schoeb, T. R. and Weaver, C. T. (2013). Allogeneic Th1 cells home to host bone marrow and spleen and mediate IFNgamma-dependent aplasia. *Biol. Blood Marrow Transplant.* 19, 876-887. 10.1016/j.bbmt.2013.03.007 23523972 PMC3683565

[DMM052084C20] Chia, R., Achilli, F., Festing, M. F. and Fisher, E. M. (2005). The origins and uses of mouse outbred stocks. *Nat. Genet.* 37, 1181-1186. 10.1038/ng1665 16254564

[DMM052084C151] Chun, S., Phan, M. T., Hong, S., Yang, J., Yoon, Y., Han, S., Kang, J., Yazer, M. H., Kim, J. and Cho, D. (2020). Double-filtered leukoreduction as a method for risk reduction of transfusion-associated graft-versus-host disease. *PLoS One* 15, e0229724. 10.1371/journal.pone.0229724 32214402 PMC7098637

[DMM052084C21] Chupp, D. P., Rivera, C. E., Zhou, Y., Xu, Y., Ramsey, P. S., Xu, Z., Zan, H. and Casali, P. (2024). A humanized mouse that mounts mature class-switched, hypermutated and neutralizing antibody responses. *Nat. Immunol.* 25, 1489-1506. 10.1038/s41590-024-01880-3 38918608 PMC11291283

[DMM052084C22] Collaborative Cross Consortium (2012). The genome architecture of the Collaborative Cross mouse genetic reference population. *Genetics* 190, 389-401. 10.1534/genetics.111.132639 22345608 PMC3276630

[DMM052084C23] Courtois, J., Ritacco, C., Dubois, S., Canti, L., Vandenhove, B., Seidel, L., Daulne, C., Caers, J., Servais, S., Beguin, Y. et al. (2021). Itacitinib prevents xenogeneic GVHD in humanized mice. *Bone Marrow. Transplant.* 56, 2672-2681. 10.1038/s41409-021-01363-1 34172892 PMC8563409

[DMM052084C24] Cuthbert, R. J., Iqbal, A., Gates, A., Toghill, P. J. and Russell, N. H. (1995). Functional hyposplenism following allogeneic bone marrow transplantation. *J. Clin. Pathol.* 48, 257-259. 10.1136/jcp.48.3.257 7730489 PMC502466

[DMM052084C152] Cuthbertson, P., Adhikary, S. R., Geraghty, N. J., Guy, T. V., Hadjiashrafi, A., Fuller, S. J., Ly, D., Watson, D. and Sluyter, R. (2020). Increased P2X7 expression in the gastrointestinal tract and skin in a humanised mouse model of graft-versus-host disease. *Clin. Sci.* 134, 207-223. 10.1042/CS20191086 31934722

[DMM052084C25] Deeg, H. J., Storb, R., Weiden, P. L., Raff, R. F., Sale, G. E., Atkinson, K., Graham, T. C. and Thomas, E. D. (1982). Cyclosporin A and methotrexate in canine marrow transplantation: engraftment, graft-versus-host disease, and induction of intolerance. *Transplantation* 34, 30-35. 10.1097/00007890-198207000-00006 6750877

[DMM052084C26] Dittmar, D. J., Pielmeier, F., Strieder, N., Fischer, A., Herbst, M., Stanewsky, H., Wenzl, N., Roseler, E., Eder, R., Gebhard, C. et al. (2024). Donor regulatory T cells rapidly adapt to recipient tissues to control murine acute graft-versus-host disease. *Nat. Commun.* 15, 3224. 10.1038/s41467-024-47575-z 38622133 PMC11018811

[DMM052084C27] Du, J., Paz, K., Thangavelu, G., Schneidawind, D., Baker, J., Flynn, R., Duramad, O., Feser, C., Panoskaltsis-Mortari, A., Negrin, R. S. et al. (2017). Invariant natural killer T cells ameliorate murine chronic GVHD by expanding donor regulatory T cells. *Blood* 129, 3121-3125. 10.1182/blood-2016-11-752444 28416503 PMC5465838

[DMM052084C28] Dudakov, J. A., Mertelsmann, A. M., O'connor, M. H., Jenq, R. R., Velardi, E., Young, L. F., Smith, O. M., Boyd, R. L., Van Den Brink, M. R. M. and Hanash, A. M. (2017). Loss of thymic innate lymphoid cells leads to impaired thymopoiesis in experimental graft-versus-host disease. *Blood* 130, 933-942. 10.1182/blood-2017-01-762658 28607133 PMC5561900

[DMM052084C29] Dutt, S., Ermann, J., Tseng, D., Liu, Y. P., George, T. I., Fathman, C. G. and Strober, S. (2005). L-selectin and beta7 integrin on donor CD4 T cells are required for the early migration to host mesenteric lymph nodes and acute colitis of graft-versus-host disease. *Blood* 106, 4009-4015. 10.1182/blood-2005-06-2339 16105972 PMC1895109

[DMM052084C156] Elhage, A., Sligar, C., Cuthbertson, P., Watson, D. and Sluyter, R. (2022). Insights into mechanisms of graft-versus-host disease through humanised mouse models. *Biosci. Rep.* 42, BSR20211986. 10.1042/BSR20211986 35993192 PMC9446388

[DMM052084C30] Enriquez, J., McDaniel Mims, B., Stroever, S., Dos Santos, A. P., Jones-Hall, Y., Furr, K. L. and Grisham, M. B. (2023). Influence of housing temperature and genetic diversity on allogeneic T cell-induced tissue damage in mice. *Pathophysiology* 30, 522-547. 10.3390/pathophysiology30040039 37987308 PMC10661280

[DMM052084C31] Enriquez, J., Mims, B. M. D., Trasti, S., Furr, K. L. and Grisham, M. B. (2020). Genomic, microbial and environmental standardization in animal experimentation limiting immunological discovery. *BMC Immunol.* 21, 50. 10.1186/s12865-020-00380-x 32878597 PMC7464063

[DMM052084C32] Ericsson, A. C., Davis, J. W., Spollen, W., Bivens, N., Givan, S., Hagan, C. E., McIntosh, M. and Franklin, C. L. (2015). Effects of vendor and genetic background on the composition of the fecal microbiota of inbred mice. *PLoS ONE* 10, e0116704. 10.1371/journal.pone.0116704 25675094 PMC4326421

[DMM052084C158] Eyrich, M., Burger, G., Marquardt, K., Budach, W., Schilbach, K., Niethammer, D. and Schlegel, P. G. (2005). Sequential expression of adhesion and costimulatory molecules in graft-versus-host disease target organs after murine bone marrow transplantation across minor histocompatibility antigen barriers. *Biol. Blood Marrow Transplant.* 11, 371-382. 10.1016/j.bbmt.2005.02.002 15846291

[DMM052084C159] Fanning, S. L., Appel, M. Y., Berger, S. A., Korngold, R. and Friedman, T. M. (2009). The immunological impact of genetic drift in the B10.BR congenic inbred mouse strain. *J. Immunol.* 183, 4261-4272. 10.4049/jimmunol.0900971 19752227

[DMM052084C33] Feng, X., Kim, J., Gonzalez-Matias, G., Aggarwal, N., Manley, A. L., Wu, Z., Solorzano, S., Batchu, S., Gao, S., Chen, J. et al. (2023). Granulocytic myeloid-derived suppressor cells to prevent and treat murine immune-mediated bone marrow failure. *Blood Adv.* 7, 73-86. 10.1182/bloodadvances.2022007254 35895513 PMC9827041

[DMM052084C34] Fox, J. G., Ge, Z., Whary, M. T., Erdman, S. E. and Horwitz, B. H. (2011). Helicobacter hepaticus infection in mice: models for understanding lower bowel inflammation and cancer. *Mucosal. Immunol.* 4, 22-30. 10.1038/mi.2010.61 20944559 PMC3939708

[DMM052084C35] Fredricks, D. N. (2019). The gut microbiota and graft-versus-host disease. *J. Clin. Invest.* 129, 1808-1817. 10.1172/JCI125797 31042160 PMC6486325

[DMM052084C36] Fu, Y. Y., Egorova, A., Sobieski, C., Kuttiyara, J., Calafiore, M., Takashima, S., Clevers, H. and Hanash, A. M. (2019). T Cell recruitment to the intestinal stem cell compartment drives immune-mediated intestinal damage after allogeneic transplantation. *Immunity* 51, 90-103.e3. 10.1016/j.immuni.2019.06.003 31278057 PMC7239328

[DMM052084C37] Gadina, M., Le, M. T., Schwartz, D. M., Silvennoinen, O., Nakayamada, S., Yamaoka, K. and O'shea, J. J. (2019). Janus kinases to jakinibs: from basic insights to clinical practice. *Rheumatology* 58, i4-i16. 10.1093/rheumatology/key432 30806710 PMC6657570

[DMM052084C38] Gao, C., Gardner, D., Theobalds, M. C., Hitchcock, S., Deutsch, H., Amuzie, C., Cesaroni, M., Sargsyan, D., Rao, T. S. and Malaviya, R. (2021). Cytotoxic T lymphocyte antigen-4 regulates development of xenogenic graft versus host disease in mice via modulation of host immune responses induced by changes in human T cell engraftment and gene expression. *Clin. Exp. Immunol.* 206, 422-438. 10.1111/cei.13659 34487545 PMC8561689

[DMM052084C161] Geraghty, N. J., Belfiore, L., Ly, D., Adhikary, S. R., Fuller, S. J., Varikatt, W., Sanderson-Smith, M. L., Sluyter, V., Alexander, S. I., Sluyter, R. et al. (2017). The P2X7 receptor antagonist Brilliant Blue G reduces serum human interferon-γ in a humanized mouse model of graft-versus-host disease. *Clin. Exp. Immunol.* 190, 79-95. 10.1111/cei.13005 28665482 PMC5588776

[DMM052084C39] Glenn, J. D. and Whartenby, K. A. (2014). Mesenchymal stem cells: Emerging mechanisms of immunomodulation and therapy. *World J. Stem Cells* 6, 526-539. 10.4252/wjsc.v6.i5.526 25426250 PMC4178253

[DMM052084C40] Graham, J. B., Thomas, S., Swarts, J., McMillan, A. A., Ferris, M. T., Suthar, M. S., Treuting, P. M., Ireton, R., Gale, M., & Lund, J. R. et al. (2015). Genetic diversity in the collaborative cross model recapitulates human West Nile virus disease outcomes. *MBio* 6, e00493-e00415. 10.1128/mBio.00493-15 25944860 PMC4436067

[DMM052084C41] Graham, J. B., Swarts, J. L., Mooney, M., Choonoo, G., Jeng, S., Miller, D. R., Ferris, M. T., McWeeney, S. and Lund, J. M. (2017). Extensive homeostatic T cell phenotypic variation within the collaborative cross. *Cell Rep.* 21, 2313-2325. 10.1016/j.celrep.2017.10.093 29166619 PMC5728448

[DMM052084C42] Grimm, D. (2021). Controversial study says U.S. labs use 111 million mice, rats. *Science* 371, 332-333. 10.1126/science.371.6527.332 33479131

[DMM052084C43] Gyurkocza, B. and Sandmaier, B. M. (2014). Conditioning regimens for hematopoietic cell transplantation: one size does not fit all. *Blood* 124, 344-353. 10.1182/blood-2014-02-514778 24914142 PMC4102707

[DMM052084C44] Haley, P. J. (2003). Species differences in the structure and function of the immune system. *Toxicology* 188, 49-71. 10.1016/S0300-483X(03)00043-X 12748041

[DMM052084C45] Hall, C. H. T. and de Zoeten, E. F. (2024). Understanding very early onset inflammatory bowel disease (VEOIBD) in relation to inborn errors of immunity. *Immunol. Rev.* 322, 329-338. 10.1111/imr.13302 38115672 PMC11044353

[DMM052084C46] Hess, N. J., Brown, M. E. and Capitini, C. M. (2021). GVHD Pathogenesis, Prevention and Treatment: Lessons From Humanized Mouse Transplant Models. *Front Immunol.* 12, 723544. 10.3389/fimmu.2021.723544 34394131 PMC8358790

[DMM052084C47] Hess, N. J., Hudson, A. W., Hematti, P. and Gumperz, J. E. (2020). Early T cell activation metrics predict graft-versus-host disease in a humanized mouse model of hematopoietic stem cell transplantation. *J. Immunol.* 205, 272-281. 10.4049/jimmunol.2000054 32444392 PMC7329317

[DMM052084C48] Hill, G. R. and Ferrara, J. L. (2000). The primacy of the gastrointestinal tract as a target organ of acute graft-versus-host disease: rationale for the use of cytokine shields in allogeneic bone marrow transplantation. *Blood* 95, 2754-2759. 10.1182/blood.V95.9.2754.009k25_2754_2759 10779417

[DMM052084C49] Hill, G. R. and Koyama, M. (2020). Cytokines and costimulation in acute graft-versus-host disease. *Blood* 136, 418-428. 10.1182/blood.2019000952 32526028 PMC7378458

[DMM052084C50] Hill, G. R., Crawford, J. M., Cooke, K. R., Brinson, Y. S., Pan, L. and Ferrara, J. L. (1997). Total body irradiation and acute graft-versus-host disease: the role of gastrointestinal damage and inflammatory cytokines. *Blood* 90, 3204-3213. 10.1182/blood.V90.8.3204 9376604

[DMM052084C51] Hill, G. R., Betts, B. C., Tkachev, V., Kean, L. S. and Blazar, B. R. (2021). Current Concepts and Advances in Graft-Versus-Host Disease Immunology. *Annu. Rev. Immunol.* 39, 19-49. 10.1146/annurev-immunol-102119-073227 33428454 PMC8085043

[DMM052084C52] Hippen, K. L., Hefazi, M., Larson, J. H. and Blazar, B. R. (2022). Emerging translational strategies and challenges for enhancing regulatory T cell therapy for graft-versus-host disease. *Front. Immunol.* 13, 926550. 10.3389/fimmu.2022.926550 35967386 PMC9366169

[DMM052084C53] Hylander, B. L., Gordon, C. J. and Repasky, E. A. (2019). Manipulation of Ambient Housing Temperature To Study the Impact of Chronic Stress on Immunity and Cancer in Mice. *J. Immunol.* 202, 631-636. 10.4049/jimmunol.1800621 30670578 PMC6352311

[DMM052084C54] Hylander, B. L., Qiao, G., Cortes Gomez, E., Singh, P. and Repasky, E. A. (2023). Housing temperature plays a critical role in determining gut microbiome composition in research mice: Implications for experimental reproducibility. *Biochimie* 210, 71-81. 10.1016/j.biochi.2023.01.016 36693616 PMC10953156

[DMM052084C55] Hylander, B. L. and Repasky, E. A. (2016). Thermoneutrality, mice, and cancer: a heated opinion. *Trends Cancer* 2, 166-175. 10.1016/j.trecan.2016.03.005 28741570

[DMM052084C157] Ito, R., Katano, I., Kawai, K., Hirata, H., Ogura, T., Kamisako, T., Eto, T. and Ito, M. (2009). Highly sensitive model for xenogenic GVHD using severe immunodeficient NOG mice. *Transplantation* 87, 1654-1658. 10.1097/TP.0b013e3181a5cb07 19502956

[DMM052084C56] Ivanov, I. I., Atarashi, K., Manel, N., Brodie, E. L., Shima, T., Karaoz, U., Wei, D., Goldfarb, K. C., Santee, C. A., Lynch, S. V. et al. (2009). Induction of intestinal Th17 cells by segmented filamentous bacteria. *Cell* 139, 485-498. 10.1016/j.cell.2009.09.033 19836068 PMC2796826

[DMM052084C57] James, C. M., Olejniczak, S. H. and Repasky, E. A. (2023). How murine models of human disease and immunity are influenced by housing temperature and mild thermal stress. *Temperature* 10, 166-178. 10.1080/23328940.2022.2093561 PMC10274546 37332306

[DMM052084C58] Johansson, J. E. and Ekman, T. (2007). Gut toxicity during hemopoietic stem cell transplantation may predict acute graft-versus-host disease severity in patients. *Dig. Dis. Sci.* 52, 2340-2345. 10.1007/s10620-006-9404-x 17415646

[DMM052084C59] Josefsdottir, K. S., Baldridge, M. T., Kadmon, C. S. and King, K. Y. (2017). Antibiotics impair murine hematopoiesis by depleting the intestinal microbiota. *Blood* 129, 729-739. 10.1182/blood-2016-03-708594 27879260 PMC5301822

[DMM052084C60] Kadri, N., Amu, S., Iacobaeus, E., Boberg, E. and Le Blanc, K. (2023). Current perspectives on mesenchymal stromal cell therapy for graft versus host disease. *Cell. Mol. Immunol.* 20, 613-625. 10.1038/s41423-023-01022-z 37165014 PMC10229573

[DMM052084C160] Kaplan, D. H., Anderson, B. E., McNiff, J. M., Jain, D., Shlomchik, M. J. and Shlomchik, W. D. (2004). Target antigens determine graft-versus-host disease phenotype. *J. Immunol.* 173, 5467-5475. 10.4049/jimmunol.173.9.5467 15494494

[DMM052084C61] Kappel, L. W., Goldberg, G. L., King, C. G., Suh, D. Y., Smith, O. M., Ligh, C., Holland, A. M., Grubin, J., Mark, N. M., Liu, C. et al. (2009). IL-17 contributes to CD4-mediated graft-versus-host disease. *Blood* 113, 945-952. 10.1182/blood-2008-08-172155 18931341 PMC2630280

[DMM052084C62] Karl, J. P., Hatch, A. M., Arcidiacono, S. M., Pearce, S. C., Pantoja-Feliciano, I. G., Doherty, L. A. and Soares, J. W. (2018). Effects of psychological, environmental and physical stressors on the gut microbiota. *Front. Microbiol.* 9, 2013. 10.3389/fmicb.2018.02013 30258412 PMC6143810

[DMM052084C63] Karp, C. L. (2012). Unstressing intemperate models: how cold stress undermines mouse modeling. *J. Exp. Med.* 209, 1069-1074. 10.1084/jem.20120988 22665703 PMC3371737

[DMM052084C64] Kawasaki, Y., Sato, K., Hayakawa, H., Takayama, N., Nakano, H., Ito, R., Mashima, K., Oh, I., Minakata, D., Yamasaki, R. et al. (2018). Comprehensive analysis of the activation and proliferation kinetics and effector functions of human lymphocytes, and antigen presentation capacity of antigen-presenting cells in xenogeneic graft-versus-host disease. *Biol. Blood Marrow Transplant.* 24, 1563-1574. 10.1016/j.bbmt.2018.04.016 29678638

[DMM052084C65] Kean, L. S., Burns, L. J., Kou, T. D., Kapikian, R., Lozenski, K., Langston, A., Horan, J. T., Watkins, B., Qayed, M., Bratrude, B. et al. (2024). Abatacept for acute graft-versus-host disease prophylaxis after unrelated donor hematopoietic cell transplantation. *Blood* 144, 1834-1845. 10.1182/blood.2023023660 39028876 PMC11530361

[DMM052084C66] Kennedy, G. A., Tey, S. K., Buizen, L., Varelias, A., Gartlan, K. H., Curley, C., Olver, S. D., Chang, K., Butler, J. P., Misra, A. et al. (2021). A phase 3 double-blind study of the addition of tocilizumab vs placebo to cyclosporin/methotrexate GVHD prophylaxis. *Blood* 137, 1970-1979. 10.1182/blood.2020009050 33512442

[DMM052084C67] Khuat, L. T., Vick, L. V., Dunai, C., Collins, C. P., More, S. K., Le, C. T., Pai, C. S., Stoffel, K. M., Maverakis, E., Canter, R. J. et al. (2021). Increased efficacy of dual proinflammatory cytokine blockade on acute GVHD while maintaining GVT effects. *Blood* 138, 2583-2588. 10.1182/blood.2021011216 34424962 PMC8678998

[DMM052084C68] Kim, S., Ruminski, P., Singh, M., Staser, K., Ashami, K., Ritchey, J., Lim, S., Dipersio, J. F. and Choi, J. (2024). Novel JAK inhibitors to reduce graft-versus-host disease after allogeneic hematopoietic cell transplantation in a preclinical mouse model. *Molecules* 29, 1801. 10.3390/molecules29081801 38675621 PMC11052071

[DMM052084C69] King, M. A., Covassin, L., Brehm, M. A., Racki, W., Pearson, T., Leif, J., Laning, J., Fodor, W., Foreman, O., Burzenski, L. et al. (2009). Human peripheral blood leucocyte non-obese diabetic-severe combined immunodeficiency interleukin-2 receptor gamma chain gene mouse model of xenogeneic graft-versus-host-like disease and the role of host major histocompatibility complex. *Clin. Exp. Immunol.* 157, 104-118. 10.1111/j.1365-2249.2009.03933.x 19659776 PMC2710598

[DMM052084C70] Kolb, H. J., Rieder, I., Rodt, H., Netzel, B., Grosse-Wilde, H., Scholz, S., Schaffer, E., Kolb, H. and Thierfelder, S. (1979). Antilymphocytic antibodies and marrow transplantation. VI. Graft-versus-host tolerance in DLA-incompatible dogs after in vitro treatment of bone marrow with absorbed antithymocyte globulin. *Transplantation* 27, 242-245. 10.1097/00007890-197904000-00007 35870

[DMM052084C71] Kriegel, M. A., Sefik, E., Hill, J. A., Wu, H. J., Benoist, C. and Mathis, D. (2011). Naturally transmitted segmented filamentous bacteria segregate with diabetes protection in nonobese diabetic mice. *Proc. Natl. Acad. Sci. USA* 108, 11548-11553. 10.1073/pnas.1108924108 21709219 PMC3136249

[DMM052084C72] Le Blanc, K. and Mougiakakos, D. (2012). Multipotent mesenchymal stromal cells and the innate immune system. *Nat. Rev. Immunol.* 12, 383-396. 10.1038/nri3209 22531326

[DMM052084C73] Lee, Y. S., Kim, T. Y., Kim, Y., Kim, S., Lee, S. H., Seo, S. U., Zhou, B. O., Eunju, O., Kim, K. S. and Kweon, M. N. (2021). Microbiota-derived lactate promotes hematopoiesis and erythropoiesis by inducing stem cell factor production from leptin receptor+ niche cells. *Exp. Mol. Med.* 53, 1319-1331. 10.1038/s12276-021-00667-y 34497346 PMC8492757

[DMM052084C74] Leigh, N. D., Kokolus, K. M., O'neill, R. E., Du, W., Eng, J. W., Qiu, J., Chen, G. L., McCarthy, P. L., Farrar, J. D., Cao, X. et al. (2015). Housing temperature-induced stress is suppressing murine graft-versus-host disease through beta2-adrenergic receptor signaling. *J. Immunol.* 195, 5045-5054. 10.4049/jimmunol.1500700 26459348 PMC4637222

[DMM052084C75] Leist, S. R. and Baric, R. S. (2018). Giving the genes a shuffle: using natural variation to understand host genetic contributions to viral infections. *Trends Genet.* 34, 777-789. 10.1016/j.tig.2018.07.005 30131185 PMC7114642

[DMM052084C76] Li, J., Dubois, W., Thovarai, V., Wu, Z., Feng, X., Peat, T., Zhang, S., Sen, S. K., Trinchieri, G., Chen, J. et al. (2020). Attenuation of immune-mediated bone marrow damage in conventionally housed mice. *Mol. Carcinog.* 59, 237-245. 10.1002/mc.23151 31898340 PMC7432962

[DMM052084C77] Lin, S., Lin, Y., Nery, J. R., Urich, M. A., Breschi, A., Davis, C. A., Dobin, A., Zaleski, C., Beer, M. A., Chapman, W. C. et al. (2014). Comparison of the transcriptional landscapes between human and mouse tissues. *Proc. Natl. Acad. Sci. USA* 111, 17224-17229. 10.1073/pnas.1413624111 25413365 PMC4260565

[DMM052084C78] Liu, Q., Pickett, T., Hodge, D., Rios, C., Arnold, M., Dong, G., Hamilton, S. E. and Rehermann, B. (2024). Leveraging dirty mice that have microbial exposure to improve preclinical models of human immune status and disease. *Nat. Immunol.* 25, 947-950. 10.1038/s41590-024-01842-9 38750319 PMC11656454

[DMM052084C79] Lo, B. C., Kryczek, I., Yu, J., Vatan, L., Caruso, R., Matsumoto, M., Sato, Y., Shaw, M. H., Inohara, N., Xie, Y. et al. (2024). Microbiota-dependent activation of CD4(+) T cells induces CTLA-4 blockade-associated colitis via Fcgamma receptors. *Science* 383, 62-70. 10.1126/science.adh8342 38175892 PMC12091338

[DMM052084C80] Malard, F., Holler, E., Sandmaier, B. M., Huang, H. and Mohty, M. (2023). Acute graft-versus-host disease. *Nat. Rev. Dis. Primers* 9, 27. 10.1038/s41572-023-00438-1 37291149

[DMM052084C81] Mapara, M. Y., Leng, C., Kim, Y. M., Bronson, R., Lokshin, A., Luster, A. and Sykes, M. (2006). Expression of chemokines in GVHD target organs is influenced by conditioning and genetic factors and amplified by GVHR. *Biol. Blood Marrow Transplant.* 12, 623-634. 10.1016/j.bbmt.2006.02.005 16737935

[DMM052084C82] Marin, N., Mecha, M., Espejo, C., Mestre, L., Eixarch, H., Montalban, X., Alvarez-Cermeno, J. C., Guaza, C. and Villar, L. M. (2014). Regulatory lymphocytes are key factors in MHC-independent resistance to EAE. *J Immunol. Res.* 2014, 156380. 10.1155/2014/156380 24868560 PMC4020375

[DMM052084C83] Markey, K. A., MacDonald, K. P. and Hill, G. R. (2014). The biology of graft-versus-host disease: experimental systems instructing clinical practice. *Blood* 124, 354-362. 10.1182/blood-2014-02-514745 24914137 PMC4102708

[DMM052084C84] Maron, R., Hancock, W. W., Slavin, A., Hattori, M., Kuchroo, V. and Weiner, H. L. (1999). Genetic susceptibility or resistance to autoimmune encephalomyelitis in MHC congenic mice is associated with differential production of pro- and anti-inflammatory cytokines. *Int. Immunol.* 11, 1573-1580. 10.1093/intimm/11.9.1573 10464178

[DMM052084C85] Martin, M. D., Danahy, D. B., Hartwig, S. M., Harty, J. T. and Badovinac, V. P. (2017). Revealing the complexity in CD8 T cell responses to infection in inbred C57B/6 versus outbred swiss mice. *Front. Immunol.* 8, 1527. 10.3389/fimmu.2017.01527 29213267 PMC5702636

[DMM052084C166] Martinov, T., McKenna, K. M., Tan, W. H., Collins, E. J., Kehret, A. R., Linton, J. D., Olsen, T. M., Shobaki, N. and Rongvaux, A. (2021). Building the next generation of humanized hemato-lymphoid system mice. *Front. Immunol*. 12, 643852. 10.3389/fimmu.2021.643852 33692812 PMC7938325

[DMM052084C86] Masopust, D., Sivula, C. P. and Jameson, S. C. (2017). Of mice, dirty mice, and men: using mice to understand human immunology. *J. Immunol.* 199, 383-388. 10.4049/jimmunol.1700453 28696328 PMC5512602

[DMM052084C87] McDaniel Mims, B., Jones-Hall, Y., Dos Santos, A. P., Furr, K., Enriquez, J. and Grisham, M. B. (2019). Induction of acute graft vs. host disease in lymphopenic mice. *Pathophysiology* 26, 233-244. 10.1016/j.pathophys.2019.06.002 31248669

[DMM052084C88] McDaniel Mims, B., Enriquez, J., Pires Dos Santos, A., Jones-Hall, Y., Dowd, S., Furr, K. L. and Grisham, M. B. (2021). Antibiotic administration exacerbates acute graft vs. host disease-induced bone marrow and spleen damage in lymphopenic mice. *PLoS ONE* 16, e0254845. 10.1371/journal.pone.0254845 34358240 PMC8346256

[DMM052084C89] Mestas, J. and Hughes, C. C. (2004). Of mice and not men: differences between mouse and human immunology. *J. Immunol.* 172, 2731-2738. 10.4049/jimmunol.172.5.2731 14978070

[DMM052084C90] Mirsoian, A., Bouchlaka, M. N., Sckisel, G. D., Chen, M., Pai, C. C., Maverakis, E., Spencer, R. G., Fishbein, K. W., Siddiqui, S., Monjazeb, A. M. et al. (2014). Adiposity induces lethal cytokine storm after systemic administration of stimulatory immunotherapy regimens in aged mice. *J. Exp. Med.* 211, 2373-2383. 10.1084/jem.20140116 25366964 PMC4235633

[DMM052084C91] Muskens, K. F., Lindemans, C. A. and Belderbos, M. E. (2021). Hematopoietic dysfunction during graft-versus-host disease: a self-destructive process? *Cells* 10, 2051. 10.3390/cells10082051 34440819 PMC8392486

[DMM052084C92] Nalle, S. C. and Turner, J. R. (2015). Intestinal barrier loss as a critical pathogenic link between inflammatory bowel disease and graft-versus-host disease. *Mucosal. Immunol.* 8, 720-730. 10.1038/mi.2015.40 25943273

[DMM052084C93] Nalle, S. C., Kwak, H. A., Edelblum, K. L., Joseph, N. E., Singh, G., Khramtsova, G. F., Mortenson, E. D., Savage, P. A. and Turner, J. R. (2014). Recipient NK cell inactivation and intestinal barrier loss are required for MHC-matched graft-versus-host disease. *Sci. Transl. Med.* 6, 243ra87. 10.1126/scitranslmed.3008941 PMC4161673 24990882

[DMM052084C94] Nguyen, T. L., Vieira-Silva, S., Liston, A. and Raes, J. (2015). How informative is the mouse for human gut microbiota research? *Dis. Model. Mech.* 8, 1-16. 10.1242/dmm.017400 25561744 PMC4283646

[DMM052084C95] Nikodemova, M. and Watters, J. J. (2011). Outbred ICR/CD1 mice display more severe neuroinflammation mediated by microglial TLR4/CD14 activation than inbred C57Bl/6 mice. *Neuroscience* 190, 67-74. 10.1016/j.neuroscience.2011.06.006 21683771 PMC3156380

[DMM052084C96] O'Donnell, P. V. and Jones, R. J. (2023). The development of post-transplant cyclophosphamide: Half a century of translational team science. *Blood Rev.* 62, 101034. 10.1016/j.blre.2022.101034 36435690 PMC11001251

[DMM052084C97] Patel, D. A., Schroeder, M. A., Choi, J. and Dipersio, J. F. (2022). Mouse models of graft-versus-host disease. *Methods Cell Biol.* 168, 41-66. 10.1016/bs.mcb.2021.12.008 35366991

[DMM052084C98] Peled, J. U., Gomes, A. L. C., Devlin, S. M., Littmann, E. R., Taur, Y., Sung, A. D., Weber, D., Hashimoto, D., Slingerland, A. E., Slingerland, J. B. et al. (2020). Microbiota as predictor of mortality in allogeneic hematopoietic-cell transplantation. *N. Engl. J. Med.* 382, 822-834. 10.1056/NEJMoa1900623 32101664 PMC7534690

[DMM052084C99] Petrovic, A., Alpdogan, O., Willis, L. M., Eng, J. M., Greenberg, A. S., Kappel, B. J., Liu, C., Murphy, G. J., Heller, G. and Van Den Brink, M. R. (2004). LPAM (alpha 4 beta 7 integrin) is an important homing integrin on alloreactive T cells in the development of intestinal graft-versus-host disease. *Blood* 103, 1542-1547. 10.1182/blood-2003-03-0957 14563643

[DMM052084C100] Phelan, R., Chen, M., Bupp, C., Bolon, Y. T., Broglie, L., Brunner-Grady, J., Burns, L. J., Chhabra, S., Christianson, D., Cusatis, R. et al. (2022). Updated trends in hematopoietic cell transplantation in the United States with an additional focus on adolescent and young adult transplantation activity and outcomes. *Transplant Cell Ther.* 28, 409.e1-409.e10. 10.1016/j.jtct.2022.04.012 PMC9840526 35447374

[DMM052084C101] Phillippi, J., Xie, Y., Miller, D. R., Bell, T. A., Zhang, Z., Lenarcic, A. B., Aylor, D. L., Krovi, S. H., Threadgill, D. W., De Villena, F. P. et al. (2014). Using the emerging Collaborative Cross to probe the immune system. *Genes Immun.* 15, 38-46. 10.1038/gene.2013.59 24195963 PMC4004367

[DMM052084C102] Pingali, S. R. and Champlin, R. E. (2015). Pushing the envelope-nonmyeloablative and reduced intensity preparative regimens for allogeneic hematopoietic transplantation. *Bone Marrow. Transplant.* 50, 1157-1167. 10.1038/bmt.2015.61 25985053 PMC4809137

[DMM052084C103] Rao, A., Kamani, N., Filipovich, A., Lee, S. M., Davies, S. M., Dalal, J. and Shenoy, S. (2007). Successful bone marrow transplantation for IPEX syndrome after reduced-intensity conditioning. *Blood* 109, 383-385. 10.1182/blood-2006-05-025072 16990602

[DMM052084C104] Rayasam, A. and Drobyski, W. R. (2021). Translational clinical strategies for the prevention of gastrointestinal tract graft versus host disease. *Front. Immunol.* 12, 779076. 10.3389/fimmu.2021.779076 34899738 PMC8662938

[DMM052084C105] Reddy, P., Negrin, R. and Hill, G. R. (2008). Mouse models of bone marrow transplantation. *Biol. Blood Marrow Transplant.* 14, 129-135. 10.1016/j.bbmt.2007.10.021 18162233 PMC2880470

[DMM052084C106] Rehermann, B., Graham, A. L., Masopust, D. and Hamilton, S. E. (2024). Integrating natural commensals and pathogens into preclinical mouse models. *Nat. Rev. Immunol*. 10.1038/s41577-024-01108-3 PMC12126596 39562646

[DMM052084C107] Reichenbach, D. K., Li, Q., Hoffman, R. A., Williams, A. L., Shlomchik, W. D., Rothstein, D. M., Demetris, A. J. and Lakkis, F. G. (2013). Allograft outcomes in outbred mice. *Am. J. Transplant.* 13, 580-588. 10.1111/ajt.12056 23311531 PMC3582712

[DMM052084C108] Webb, R., Den Bakker, C., Koboziev, H., Jones-Hall, I., Rao Kottapalli, Y., Ostanin, K., Furr, D., Mu, K. L., Luo, Q., & Grisham, X. M. et al. (2018). Differential susceptibility to T cell-induced colitis in mice: role of the intestinal microbiota. *Inflamm. Bowel Dis.* 24, 361-379. 10.1093/ibd/izx014 29361089 PMC6176899

[DMM052084C109] Riegel, C., Boeld, T. J., Doser, K., Huber, E., Hoffmann, P. and Edinger, M. (2020). Efficient treatment of murine acute GvHD by in vitro expanded donor regulatory T cells. *Leukemia* 34, 895-908. 10.1038/s41375-019-0625-3 31719679 PMC7214258

[DMM052084C110] Rongvaux, A., Takizawa, H., Strowig, T., Willinger, T., Eynon, E. E., Flavell, R. A. and Manz, M. G. (2013). Human hemato-lymphoid system mice: current use and future potential for medicine. *Annu. Rev. Immunol.* 31, 635-674. 10.1146/annurev-immunol-032712-095921 23330956 PMC4120191

[DMM052084C111] Rongvaux, A., Willinger, T., Martinek, J., Strowig, T., Gearty, S. V., Teichmann, L. L., Saito, Y., Marches, F., Halene, S., Palucka, A. K. et al. (2014). Development and function of human innate immune cells in a humanized mouse model. *Nat. Biotechnol.* 32, 364-372. 10.1038/nbt.2858 24633240 PMC4017589

[DMM052084C112] Rosser, E. C. and Mauri, C. (2015). Regulatory B cells: origin, phenotype, and function. *Immunity* 42, 607-612. 10.1016/j.immuni.2015.04.005 25902480

[DMM052084C113] Rosser, E. C. and Mauri, C. (2021). The emerging field of regulatory B cell immunometabolism. *Cell Metab.* 33, 1088-1097. 10.1016/j.cmet.2021.05.008 34077716

[DMM052084C114] Rosshart, S. P., Vassallo, B. G., Angeletti, D., Hutchinson, D. S., Morgan, A. P., Takeda, K., Hickman, H. D., McCulloch, J. A., Badger, J. H., Ajami, N. J. et al. (2017). Wild mouse gut microbiota promotes host fitness and improves disease resistance. *Cell* 171, 1015-1028.e13. 10.1016/j.cell.2017.09.016 29056339 PMC6887100

[DMM052084C115] Saha, A., Hyzy, S., Lamothe, T., Hammond, K., Clark, N., Lanieri, L., Bhattarai, P., Palchaudhuri, R., Gillard, G. O., Proctor, J. et al. (2022). A CD45-targeted antibody-drug conjugate successfully conditions for allogeneic hematopoietic stem cell transplantation in mice. *Blood* 139, 1743-1759. 10.1182/blood.2021012366 34986233 PMC8931510

[DMM052084C116] Saha, A., Palchaudhuri, R., Lanieri, L., Hyzy, S., Riddle, M. J., Panthera, J., Eide, C. R., Tolar, J., Panoskaltsis-Mortari, A., Gorfinkel, L. et al. (2024). Alloengraftment without significant toxicity or GVHD in CD45 antibody-drug conjugate-conditioned Fanconi anemia mice. *Blood* 143, 2201-2216. 10.1182/blood.2023023549 38447038 PMC11143525

[DMM052084C117] Schroeder, M. A., Choi, J., Staser, K. and Dipersio, J. F. (2018). The role of janus kinase signaling in graft-versus-host disease and graft versus leukemia. *Biol. Blood Marrow Transplant.* 24, 1125-1134. 10.1016/j.bbmt.2017.12.797 29289756 PMC5993569

[DMM052084C118] Schroeder, M. A. and DiPersio, J. F. (2011). Mouse models of graft-versus-host disease: advances and limitations. *Dis. Model. Mech.* 4, 318-333. 10.1242/dmm.006668 21558065 PMC3097454

[DMM052084C119] Schwab, L., Goroncy, L., Palaniyandi, S., Gautam, S., Triantafyllopoulou, A., Mocsai, A., Reichardt, W., Karlsson, F. J., Radhakrishnan, S. V., Hanke, K. et al. (2014). Neutrophil granulocytes recruited upon translocation of intestinal bacteria enhance graft-versus-host disease via tissue damage. *Nat. Med.* 20, 648-654. 10.1038/nm.3517 24836575

[DMM052084C120] Scroggins, S. M. and Schlueter, A. J. (2023). Generation of human regulatory dendritic cells from cryopreserved healthy donor cells and hematopoietic stem cell transplant recipients. *Cells* 12, 2372. 10.3390/cells12192372 37830587 PMC10571850

[DMM052084C121] Shaffer, B. C., Gooptu, M., Defor, T. E., Maiers, M., Bolanos-Meade, J., Abboud, R., Briggs, A. D., Khimani, F., Modi, D., Newcomb, R. et al. (2024). Post-transplant cyclophosphamide-based graft-versus-host disease prophylaxis attenuates disparity in outcomes between use of matched or mismatched unrelated donors. *J. Clin. Oncol.* 388, 2338-2348. 10.1200/JCO.24.00184 PMC11421565 39018507

[DMM052084C122] Shahin, K., Mattar, Z., Silveira, P., Hsu, W. H., Bendall, L., Hart, D. and Bradstock, K. F. (2017). Bone marrow graft-versus-host disease in major histocompatibility complex-matched murine reduced-intensity allogeneic hemopoietic cell transplantation. *Transplantation* 101, 2695-2704. 10.1097/TP.0000000000001733 28319565

[DMM052084C123] Shaw, B. E., Jimenez-Jimenez, A. M., Burns, L. J., Logan, B. R., Khimani, F., Shaffer, B. C., Shah, N. N., Mussetter, A., Tang, X. Y., Mccarty, J. M. et al. (2021). National marrow donor program-sponsored multicenter, phase II trial of HLA-mismatched unrelated donor bone marrow transplantation using post-transplant cyclophosphamide. *J. Clin. Oncol.* 39, 1971-1982. 10.1200/JCO.20.03502 33905264 PMC8260905

[DMM052084C124] Shay, T., Jojic, V., Zuk, O., Rothamel, K., Puyraimond-Zemmour, D., Feng, T., Wakamatsu, E., Benoist, C., Koller, D., Regev, A. et al. (2013). Conservation and divergence in the transcriptional programs of the human and mouse immune systems. *Proc. Natl. Acad. Sci. USA* 110, 2946-2951. 10.1073/pnas.1222738110 23382184 PMC3581886

[DMM052084C125] Shimoni, A., Labopin, M., Savani, B., Volin, L., Ehninger, G., Kuball, J., Bunjes, D., Schaap, N., Vigouroux, S., Bacigalupo, A. et al. (2016). Long-term survival and late events after allogeneic stem cell transplantation from HLA-matched siblings for acute myeloid leukemia with myeloablative compared to reduced-intensity conditioning: a report on behalf of the acute leukemia working party of European group for blood and marrow transplantation. *J Hematol. Oncol.* 9, 118. 10.1186/s13045-016-0347-1 27821187 PMC5100212

[DMM052084C126] Shono, Y., Ueha, S., Wang, Y., Abe, J., Kurachi, M., Matsuno, Y., Sugiyama, T., Nagasawa, T., Imamura, M. and Matsushima, K. (2010). Bone marrow graft-versus-host disease: early destruction of hematopoietic niche after MHC-mismatched hematopoietic stem cell transplantation. *Blood* 115, 5401-5411. 10.1182/blood-2009-11-253559 20354171

[DMM052084C127] Shono, Y., Shiratori, S., Kosugi-Kanaya, M., Ueha, S., Sugita, J., Shigematsu, A., Kondo, T., Hashimoto, D., Fujimoto, K., Endo, T. et al. (2014). Bone marrow graft-versus-host disease: evaluation of its clinical impact on disrupted hematopoiesis after allogeneic hematopoietic stem cell transplantation. *Biol. Blood Marrow Transplant.* 20, 495-500. 10.1016/j.bbmt.2013.12.568 24374213

[DMM052084C128] Shono, Y., Docampo, M. D., Peled, J. U., Perobelli, S. M., Velardi, E., Tsai, J. J., Slingerland, A. E., Smith, O. M., Young, L. F., Gupta, J. et al. (2016). Increased GVHD-related mortality with broad-spectrum antibiotic use after allogeneic hematopoietic stem cell transplantation in human patients and mice. *Sci Transl. Med.* 8, 339ra71. 10.1126/scitranslmed.aaf2311 PMC4991773 27194729

[DMM052084C129] Spellman, S. R. (2022). Hematology 2022-what is complete HLA match in 2022? *Hematology Am. Soc. Hematol. Educ. Program* 2022, 83-89. 10.1182/hematology.2022000326 36485162 PMC9821192

[DMM052084C164] Sprent, J., Schaefer, M., Lo D. and Korngold, R. (1986). Properties of purified T cell subsets. II. In vivo responses to class I vs. class II H-2 differences. *J. Exp. Med.* 163, 998-1011. 10.1084/jem.163.4.998 3512763 PMC2188064

[DMM052084C130] Staffas, A., Burgos Da Silva, M. and Van Den Brink, M. R. (2017). The intestinal microbiota in allogeneic hematopoietic cell transplant and graft-versus-host disease. *Blood* 129, 927-933. 10.1182/blood-2016-09-691394 27940475 PMC5324712

[DMM052084C131] Stepankova, R., Powrie, F., Kofronova, O., Kozakova, H., Hudcovic, T., Hrncir, T., Uhlig, H., Read, S., Rehakova, Z., Benada, O. et al. (2007). Segmented filamentous bacteria in a defined bacterial cocktail induce intestinal inflammation in SCID mice reconstituted with CD45RBhigh CD4+ T cells. *Inflamm. Bowel. Dis.* 13, 1202-1211. 10.1002/ibd.20221 17607724

[DMM052084C132] Stolfi, J. L., Pai, C. C. and Murphy, W. J. (2016). Preclinical modeling of hematopoietic stem cell transplantation - advantages and limitations. *FEBS J.* 283, 1595-1606. 10.1111/febs.13612 26640088

[DMM052084C133] Taylor, P. A., Lees, C. J. and Blazar, B. R. (2002). The infusion of ex vivo activated and expanded CD4(+)CD25(+) immune regulatory cells inhibits graft-versus-host disease lethality. *Blood* 99, 3493-3499. 10.1182/blood.V99.10.3493 11986199

[DMM052084C134] Teshima, T. and Hill, G. R. (2021). The pathophysiology and treatment of graft-versus-host disease: lessons learnt from animal models. *Front. Immunol.* 12, 715424. 10.3389/fimmu.2021.715424 34489966 PMC8417310

[DMM052084C135] Thomas, E., Storb, R., Clift, R. A., Fefer, A., Johnson, F. L., Neiman, P. E., Lerner, K. G., Glucksberg, H. and Buckner, C. D. (1975a). Bone-marrow transplantation (first of two parts). *N. Engl. J. Med.* 292, 832-843. 10.1056/NEJM197504172921605 234595

[DMM052084C136] Thomas, E. D., Storb, R., Clift, R. A., Fefer, A., Johnson, L., Neiman, P. E., Lerner, K. G., Glucksberg, H. and Buckner, C. D. (1975b). Bone-marrow transplantation (second of two parts). *N. Engl. J. Med.* 292, 895-902. 10.1056/NEJM197504242921706 235092

[DMM052084C137] Threadgill, D. W. and Churchill, G. A. (2012). Ten years of the collaborative cross. *Genetics* 190, 291-294. 10.1534/genetics.111.138032 22345604 PMC3276648

[DMM052084C162] van Leeuwen, L., Guiffre, A., Atkinson, K., Rainer, S. P. and Sewell, W. A. (2002). A two-phase pathogenesis of graft-versus-host disease in mice. *Bone Marrow Transplant.* 29, 151-158. 10.1038/sj.bmt.1703328 11850710

[DMM052084C138] Van Lier, Y. F., Vos, J., Blom, B. and Hazenberg, M. D. (2023). Allogeneic hematopoietic cell transplantation, the microbiome, and graft-versus-host disease. *Gut. Microbes.* 15, 2178805. 10.1080/19490976.2023.2178805 36794370 PMC9980553

[DMM052084C139] Waldman, A. D., Fritz, J. M. and Lenardo, M. J. (2020). A guide to cancer immunotherapy: from T cell basic science to clinical practice. *Nat. Rev. Immunol.* 20, 651-668. 10.1038/s41577-020-0306-5 32433532 PMC7238960

[DMM052084C140] Wuttisarnwattana, P., Eid, S., Wilson, D. L. and Cooke, K. R. (2023). Assessment of therapeutic role of mesenchymal stromal cells in mouse models of graft-versus-host disease using cryo-imaging. *Sci. Rep.* 13, 1698. 10.1038/s41598-023-28478-3 36717650 PMC9886911

[DMM052084C141] Yang, I., Eibach, D., Kops, F., Brenneke, B., Woltemate, S., Schulze, J., Bleich, A., Gruber, A. D., Muthupalani, S., Fox, J. G. et al. (2013). Intestinal microbiota composition of interleukin-10 deficient C57BL/6J mice and susceptibility to Helicobacter hepaticus-induced colitis. *PLoS ONE* 8, e70783. 10.1371/journal.pone.0070783 23951007 PMC3739778

[DMM052084C163] Ye, W. and Chen, Q. (2022). Potential applications and perspectives of humanized mouse models. *Annu. Rev. Anim. Biosci.* 10, 395-417. 10.1146/annurev-animal-020420-033029 34758273

[DMM052084C142] You-Ten, K. E., Seemayer, T. A., Wisse, B., Bertley, F. M. and Lapp, W. S. (1995). Induction of a glucocorticoid-sensitive F1-anti-parental mechanism that affects engraftment during graft-versus-host disease. *J. Immunol.* 155, 172-180. 10.4049/jimmunol.155.1.172 7602093

[DMM052084C143] Zeiser, R. and Blazar, B. R. (2016). Preclinical models of acute and chronic graft-versus-host disease: how predictive are they for a successful clinical translation? *Blood* 127, 3117-3126. 10.1182/blood-2016-02-699082 26994149 PMC4920018

[DMM052084C144] Zeiser, R. and Blazar, B. R. (2017a). Acute graft-versus-host disease - biologic process, prevention, and therapy. *N. Engl. J. Med.* 377, 2167-2179. 10.1056/NEJMra1609337 29171820 PMC6034180

[DMM052084C145] Zeiser, R. and Blazar, B. R. (2017b). Pathophysiology of chronic graft-versus-host disease and therapeutic targets. *N. Engl. J. Med.* 377, 2565-2579. 10.1056/NEJMra1703472 29281578

[DMM052084C146] Zeiser, R. and Teshima, T. (2021). Nonclassical manifestations of acute GVHD. *Blood* 138, 2165-2172. 10.1182/blood.2021012431 34482399

[DMM052084C147] Zeiser, R., Socie, G. and Blazar, B. R. (2016). Pathogenesis of acute graft-versus-host disease: from intestinal microbiota alterations to donor T cell activation. *Br. J. Haematol.* 175, 191-207. 10.1111/bjh.14295 27619472

[DMM052084C148] Zeiser, R., Von Bubnoff, N., Butler, J., Mohty, M., Niederwieser, D., Or, R., Szer, J., Wagner, E. M., Zuckerman, T., Mahuzier, B. et al. (2020). Ruxolitinib for glucocorticoid-refractory acute graft-versus-host disease. *N. Engl. J. Med.* 382, 1800-1810. 10.1056/NEJMoa1917635 32320566

[DMM052084C149] Zeiser, R., Ringden, O., Sadeghi, B., Gonen-Yaacovi, G. and Segurado, O. G. (2023). Novel therapies for graft versus host disease with a focus on cell therapies. *Front. Immunol.* 14, 1241068. 10.3389/fimmu.2023.1241068 37868964 PMC10585098

[DMM052084C150] Zeng, M. Y., Inohara, N. and Nunez, G. (2017). Mechanisms of inflammation-driven bacterial dysbiosis in the gut. *Mucosal. Immunol.* 10, 18-26. 10.1038/mi.2016.75 27554295 PMC5788567

[DMM052084C165] Zhang, Y., Joe, G., Hexner, E., Zhu J. and Emerson, S. G. (2005). Alloreactive memory T cells are responsible for the persistence of graft-versus-host disease. *J. Immunol.* 174, 3051-3058. 10.4049/jimmunol.174.5.3051 15728519

